# Supplementary material for: Translating digital healthcare to enhance clinical management: a protocol for an observational study using a digital health technology system to monitor medication adherence and its effect on mobility in people with Parkinson’s
Source: BMJ Open. 2023 Sep 4;13(9):e073388. doi: 10.1136/bmjopen-2023-073388 (PMC10481731; doi:10.1136/bmjopen-2023-073388)
Supplement: Supplementary data [file bmjopen-2023-073388supp002.pdf]

CiC – Effect of medication on mobility in people with PD
Consent Form V2.1 – 14 May 2021
IRAS ID: 295771

NHS
The Newcastle upon Tyne Hospitals
NHS Foundation Trust

Principal Investigator: Dr Silvia Del Din

Participant Identification Number:

Participant Consent Form

Confidence in Concept (CiC) - Translating digital healthcare to enhance clinical management: evaluating the effect of medication on mobility in people with Parkinson’s disease (PD).

We are inviting you to take part in a medical research study.
Please make sure you have read the accompanying Participant Information Sheet which explains why we are doing this research and what we are asking you to do.
Please ask a member of the research team if there is anything that is not clear or if you would like more information.
It is entirely up to you if would like to take part or not.

Healthcare at its best
with people at our heart

CiC – Effect of medication on mobility in people with PD
Consent Form V2.1 – 14 May 2021
IRAS ID: 295771

NHS
The Newcastle upon Tyne Hospitals
NHS Foundation Trust

	Please initial box
I confirm that I have read and understand the Participant Information Sheet (version and date ……………………) for the above study. I have had the opportunity to consider the information, ask questions and have had these answered satisfactorily.	
I understand that my participation is voluntary and that I am free to withdraw at any time without giving any reason, without my medical care or legal rights being affected.	
I understand that relevant sections of my medical notes and data collected during the study may be looked at by individuals from local clinical care team, from Newcastle University, from regulatory authorities or from the NHS Trust, where it is relevant to my taking part in this research. I give permission for these individuals to have access to my records.	
I understand that the anonymised information collected about me will be stored and used to support other research in the future, and may be shared anonymously with other researchers.	
In the event that a new potential medical problem is found as a result of my participation, I agree that the research team may inform my GP or relevant medical professional about this following a discussion with myself.	
I understand that I will be provided with a wearable technology system comprising of an activity monitor, a smartwatch and a smartphone which will be worn and used for seven days following the visit.	
I agree to participate in this study.	

Name of Participant	Date	Signature
Name of Person Taking Consent	Date	Signature

Healthcare at its best
with people at our heart

NHS
The Newcastle upon Tyne Hospitals
NHS Foundation Trust

Please ensure that a copy is offered to the participant or relative/carer and record whether this offer was accepted/ declined.

Copy given [ ]	Copy declined [ ]

Original to be filed in the Trial Master File. Copy to be included in the participant medical records.

Healthcare at its best
with people at our heart
