# Supplementary material for: Translating digital healthcare to enhance clinical management: a protocol for an observational study using a digital health technology system to monitor medication adherence and its effect on mobility in people with Parkinson’s
Source: BMJ Open. 2023 Sep 4;13(9):e073388. doi: 10.1136/bmjopen-2023-073388 (PMC10481731; doi:10.1136/bmjopen-2023-073388)
Supplement: Supplementary data [file bmjopen-2023-073388supp004.pdf]

| **CiC – Effect of medication on mobility in people with PD**<br>Version 1.1; 14 May 2021; IRAS ID: 295771 | |
|---|---|
| **Subject Initial**<br>\|__\|\|__\|<br>F S | **Subject ID**<br>\|__\|__\|__\|__\|__\| |

| **Day/ Time** | **6:00<br>7:00** | **7:00<br>8:00** | **8:00<br>9:00** | **9:00<br>10:00** | **10:00<br>11:00** | **11:00<br>12:00** | **12:00<br>13:00** | **13:00<br>14:00** | **14:00<br>15:00** | **15:00<br>16:00** | **16:00<br>17:00** | **17:00<br>18:00** | **18:00<br>19:00** | **19:00<br>20:00** | **20:00<br>21:00** | **21:00<br>22:00** | Comments |
|---|---|---|---|---|---|---|---|---|---|---|---|---|---|---|---|---|---|
| Monday | | | | | | | | | | | | | | | | | |
| Tuesday | | | | | | | | | | | | | | | | | |
| Wednesday | | | | | | | | | | | | | | | | | |
| Thursday | | | | | | | | | | | | | | | | | |
| Friday | | | | | | | | | | | | | | | | | |
| Saturday | | | | | | | | | | | | | | | | | |
| Sunday | | | | | | | | | | | | | | | | | |

*Split cells indicate the "half an hour" – e.g. 6.30

Example: Off status ("O") between 6.30 and 7.00 and dyskinesia ("D") between 7.00 and 7.30 on Monday

| **Day/ Time** | **6:00<br>7:00** | | **7:00<br>8:00** | | **8:00<br>9:00** | |
|---|---|---|---|---|---|---|
| Monday | | O | D | | | |
