# Supplementary material for: Translating digital healthcare to enhance clinical management: a protocol for an observational study using a digital health technology system to monitor medication adherence and its effect on mobility in people with Parkinson’s
Source: BMJ Open. 2023 Sep 4;13(9):e073388. doi: 10.1136/bmjopen-2023-073388 (PMC10481731; doi:10.1136/bmjopen-2023-073388)
Supplement: Supplementary data [file bmjopen-2023-073388supp006.pdf]

Supplementary material 6. Table summarising the standardised and secure data transfer pipeline.

Data type	Data pipeline
**Clinical and Demographic data**	Data will be collected on paper forms, which will be scanned and uploaded to a secured password protected University drive. Processed data will be entered onto the database. Paper forms will be stored in the Gait Laboratory at the Clinical Ageing Research Unit, with access restricted to the laboratory research team.
**Mobility data from IMU**	When using the Axivity AX6 sensor, raw data will be directly uploaded to the data management platform on e-SC from the clinical site.
**Self-reported medication adherence data (smartwatch and smartphone)**	Data that are logged on the smartphone will be transferred to the University of Sheffield (USFD) servers. Processed data (e.g., acknowledged medication intake times) will then be transferred from USFD servers and uploaded to eSC platform via a secured system (e.g. API or web portal).
**Contextual data from smartphone**	Data will be transferred from the smartphone to the USFD servers. Processed data (annotations) will be transferred from USFD servers and uploaded to the eSC platform via a secured system (e.g. API or web portal).
**Usability questionnaire, open-text questions, and motor complications diary**	Usability questionnaires, responses to open-text questions, and diary data will be collected with paper forms. These forms will be scanned and uploaded to a secured password protected University drive. Processed data will be entered onto a database.
