# Supplementary material for: Translating digital healthcare to enhance clinical management: a protocol for an observational study using a digital health technology system to monitor medication adherence and its effect on mobility in people with Parkinson’s
Source: BMJ Open. 2023 Sep 4;13(9):e073388. doi: 10.1136/bmjopen-2023-073388 (PMC10481731; doi:10.1136/bmjopen-2023-073388)
Supplement: Author's manuscript [file bmjopen-2023-073388.draft_revisions.pdf]

BMJ Open is committed to open peer review. As part of this commitment we make the peer review history of every article we publish publicly available.

When an article is published we post the peer reviewers' comments and the authors' responses online. We also post the versions of the paper that were used during peer review. These are the versions that the peer review comments apply to.

The versions of the paper that follow are the versions that were submitted during the peer review process. They are not the versions of record or the final published versions. They should not be cited or distributed as the published version of this manuscript.

BMJ Open is an open access journal and the full, final, typeset and author-corrected version of record of the manuscript is available on our site with no access controls, subscription charges or pay-per-view fees (http://bmjopen.bmj.com).

If you have any questions on BMJ Open's open peer review process please email info.bmjopen@bmj.com

BMJ Open

Translating digital healthcare to enhance clinical management: evaluating the effect of medication on mobility in people with Parkinson's disease (PD) - an observational study protocol

Journal:	*BMJ Open*
Manuscript ID	bmjopen-2023-073388
Article Type:	Protocol
Date Submitted by the Author:	06-Mar-2023
Complete List of Authors:	Packer, Emma; Newcastle University Translational and Clinical Research Institute, Faculty of Medical Sciences<br>Debelle, Héloïse; Newcastle University Translational and Clinical Research Institute, Faculty of Medical Sciences<br>Bailey, Harry; Newcastle University Translational and Clinical Research Institute, Faculty of Medical Sciences<br>Ciravegna, Fabio; Università degli Studi di Torino Dipartimento di informatica<br>Ireson, Neil; The University of Sheffield, Department of Computer Science and Insigneo Institute<br>Evers, Jordi; McRoberts BV<br>Niessen, Martjin; McRoberts BV<br>Shi, Jian Qing; Southern University of Science and Technology; National Center for Applied Mathematics<br>Yarnall, Alison J; Newcastle Upon Tyne Hospitals NHS Foundation Trust; Newcastle University Translational and Clinical Research Institute, Faculty of Medical Sciences<br>Rochester, Lynn; Newcastle Upon Tyne Hospitals NHS Foundation Trust; Newcastle University Translational and Clinical Research Institute, Faculty of Medical Sciences<br>Alcock, Lisa; Newcastle University Translational and Clinical Research Institute, Faculty of Medical Sciences; NIHR Newcastle Biomedical Research Centre, based at The Newcastle upon Tyne Hospitals NHS Foundation Trust, Newcastle University<br>Del Din, Silvia; Newcastle University Translational and Clinical Research Institute, Faculty of Medical Sciences; NIHR Newcastle Biomedical Research Centre, based at The Newcastle upon Tyne Hospitals NHS Foundation Trust, Newcastle University
Keywords:	Parkinson-s disease < NEUROLOGY, Quality of Life, Protocols & guidelines < HEALTH SERVICES ADMINISTRATION & MANAGEMENT

For peer review only - http://bmjopen.bmj.com/site/about/guidelines.xhtml

For peer review only - http://bmjopen.bmj.com/site/about/guidelines.xhtml

Translating digital healthcare to enhance clinical management: evaluating the effect of medication on mobility in people with Parkinson's disease (PD) - an observational study protocol

Emma Packer (0000-0002-0491-3726), Héloïse Debelle (0000-0001-9455-7448), Harry G.B Bailey (0000-0002-4442-293X), Fabio Ciravegna (0000-0001-5817-4810), Neil Ireson (0009-0009-2278-5180), Jordi Evers (0000-0002-7970-8575), Martijn Niessen (0000-0003-3515-4731), Jian Qing Shi (0000-0002-2924-1137), Alison J Yarnall (0000-0002-3126-9163), Lynn Rochester (0000-0001-5774-9272), Lisa Alcock (0000-0002-8364-9803), Silvia Del Din* (0000-0003-1154-4751)

Translational and Clinical Research Institute, Faculty of Medical Sciences, Newcastle University, Newcastle upon Tyne, UK, Emma Packer, PhD Student

Translational and Clinical Research Institute, Faculty of Medical Sciences, Newcastle University, Newcastle upon Tyne, UK, Héloïse Debelle, Research Assistant

Translational and Clinical Research Institute, Faculty of Medical Sciences, Newcastle University, Newcastle upon Tyne, UK, Harry G.B Bailey, Research Assistant

Dipartimento di Informatica, Univsersità di Torino, Italy, Fabio Ciravegna, Professor of Pervasive Technologies

Department of Computer Science and INSIGNEO Institute for in silico Medicine, The University of Sheffield, Sheffield, UK, Neil Ireson, Research Associate

McRoberts BV, The Hague, The Netherlands, Jordi Evers, Chief Marketing Officer

McRoberts BV, The Hague, The Netherlands, Martijn Niessen, Chief Executive Officer

Department of Statistics and Data Science, Southern University of Science and Technology, Shenzhen, China | National Center for Applied Mathematics Shenzhen, China, Jian Qing Shi, Professor of Statistics

Translational and Clinical Research Institute, Faculty of Medical Sciences, Newcastle University, Newcastle upon Tyne, UK | The Newcastle Upon Tyne Hospitals NHS Foundation Trust, Newcastle upon Tyne, UK, Alison J Yarnall, NIHR BRC Clinical Senior Lecturer and Honorary Consultant in Older People's Medicine

Translational and Clinical Research Institute, Faculty of Medical Sciences, Newcastle University, Newcastle upon Tyne, UK | The Newcastle Upon Tyne Hospitals NHS Foundation Trust, Newcastle upon Tyne, UK, Lynn Rochester, Professor of Human Movement Science

Translational and Clinical Research Institute, Faculty of Medical Sciences, Newcastle University, Newcastle upon Tyne, UK | National Institute for Health and Care Research (NIHR) Newcastle Biomedical Research Centre (BRC), based at Newcastle upon Tyne Hospitals NHS Foundation Trust, Newcastle University and the Cumbria, Northumberland and Tyne and Wear (CNTW) NHS Foundation Trust, Lisa Alcock, Senior Research Associate

For peer review only - http://bmjopen.bmj.com/site/about/guidelines.xhtml

Translational and Clinical Research Institute, Faculty of Medical Sciences, Newcastle University, Newcastle upon Tyne, UK | National Institute for Health and Care Research (NIHR) Newcastle Biomedical Research Centre (BRC), based at Newcastle upon Tyne Hospitals NHS Foundation Trust, Newcastle University and the Cumbria, Northumberland and Tyne and Wear (CNTW) NHS Foundation Trust, Silvia Del Din, Newcastle University Academic Track Fellow

* Corresponding author

Corresponding author

Dr Silvia Del Din

Translational and Clinical Research Institute

Faculty of Medical Sciences

The Catalyst

3 Science Square, Room 3.27

NE4 5TG

Email: Silvia.Del-Din@newcastle.ac.uk

Phone: +44 (0) 191 208 1131

For peer review only

For peer review only - http://bmjopen.bmj.com/site/about/guidelines.xhtml

Word count: 3930 (excluding title page, abstract, contributions, acknowledgements, references, figures and tables)

Key words: Wearable Technology, Parkinson's disease, Protocol, Human, Walking, Medication Adherence.

ABSTRACT

Introduction: In Parkinson's disease (PD), impaired mobility is associated with an increased falls risk. Dopaminergic medication is typically prescribed to people with PD (PwP) to improve mobility, but complex regimens result in sub-optimal adherence. Understanding the medication habits of PwP and identifying their impact on mobility will provide essential insights to optimise medication regimens and improve mobility. However, this is typically assessed in controlled environments, during one-off clinical assessments. Digital health technology (DHT) presents a means to overcome this, by continuously and remotely monitoring mobility and medication adherence. This study aims to assess self-reported medication adherence, quantify digital mobility outcomes (DMOs), and model and predict DMOs response to medication using a novel DHT system (DHTS) (comprising a smartphone, smartwatch, and Inertial Measurement Unit (IMU)).

Methods and analysis: This study will recruit 55 participants with PD who will complete a range of general, clinical, PD-specific, and physical assessments. Participants will interact with a DHTS over seven consecutive days, to assess self-reported medication adherence, and monitor mobility and contextual factors in the real-world. Participants will complete a motor complications diary (ON-OFF-Dyskinesia) throughout the monitoring period and at the end, a questionnaire and series of open-text questions to evaluate DHTS usability. Feasibility of the DHTS and the motor complications diary will be assessed. Validated algorithms will quantify DMOs from IMU extracted walking activity. Time series modelling and deep learning techniques will model and predict DMO response to medication and assess potential effects of contextual factors. This study will provide essential insights to understand the effect of medication on real-world mobility in PwP, with the aim to optimise medication regimens and improve mobility.

Ethics and dissemination: Ethical approval was granted by London - 142 Westminster Research Ethics Committee (REC: 21/PR/0469), protocol v2.4. Results will be published in peer-reviewed journals.

Trial registration number: ISRCTN (13156149)

Article Summary

Strengths and limitations of this study

- This protocol has been developed by a multidisciplinary team of experienced clinicians, researchers, and data analysts.
- This study will monitor mobility and assess self-reported medication adherence in the real-world, enhancing the ecological validity of results.
- If efficacious, this protocol may be implemented in clinical settings to optimise medication regimens and enhance the quality of life for people with Parkinson's disease.
- Participants will only be required to attend a single clinic-based assessment, with the remainder of the study conducted in their home or community.
- This study will include participants with mild to moderate disease severity, therefore, our conclusions may not reflect the experience of those with more advanced Parkinson's disease.

For peer review only - http://bmjopen.bmj.com/site/about/guidelines.xhtml

INTRODUCTION

Mobility, defined as the ability to freely move about one's environment, is a key contributor to overall health and maintaining independence (1, 2). People with Parkinson's disease (PwP) experience a loss of mobility due to the cardinal motor symptoms of bradykinesia, rigidity, tremor, and postural instability (3). This loss of mobility is associated with an increased risk of falls and reduced independence; therefore, mitigating mobility loss in PwP is of primary importance (4).

In PwP, dopaminergic medications (e.g. Levodopa) are commonly prescribed to alleviate motor symptoms and improve mobility (3). Medication is often prescribed in multiple doses throughout the day, with adherence to prescribed dosage and timing essential to alleviate motor symptoms and avoid fluctuations, especially as the disease progresses. Complex medication regimens result in suboptimal adherence, poor medication responses, and reduced quality of life (5-7). Modification to regimens often follows patient-reported outcomes and short, infrequent clinical appointments, with clinicians observing patients during specific stages in their regimen (e.g., ON or OFF periods). Consequently, clinicians often lack adequate insight into patient's medication habits and daily fluctuations in motor symptoms to appropriately adapt regimens. Understanding medication adherence in PwP and its effect on their everyday mobility and motor symptoms would ensure more effective and personalised patient management, allowing clinicians to optimise dose and frequency of dopaminergic drugs.

At present, research studies investigating medication and mobility in PwP often include assessments performed in controlled environments such as those in lab-based or clinical environments and those performed ON or OFF medication (8-16). Although some studies have been performed in the real-world (i.e., daily living, outside clinical or lab settings) (17, 18), a more granular picture is required. Digital health technology (DHT) presents the tools to explore this, by allowing for the continuous assessment of medication adherence and mobility (digital mobility outcomes, DMOs) in the real-world (5, 19-22). A variety of DMOs can be utilised to assess mobility; and of primary interest is walking speed (23). Walking speed reflects overall health in PwP and declines overtime, with slow walking speed associated with adverse clinical events such as an increased risk of falls (4); therefore, assessing this DMO can provide insights into medication related changes in overall mobility and health. The COVID-19 pandemic has also highlighted the fragility of clinic-based care for PwP and reinforced the necessity to utilise DHT to improve current practice (24).

There are a variety of DHTs which can monitor individuals during their day-to-day lives. For example, the Personal KineticGraph (PKG, Global kinetics Corp, Australia), a wrist-worn device, can send medication reminders and continuously monitor motor symptoms such as bradykinesia, tremor, and dyskinesia (25, 26). However, technology such as this cannot monitor the specific medication ingested or quantify gait characteristics. Future research should therefore focus on developing a DHT system (DHTS) which can comprehensively and remotely assess medication adherence and monitor mobility. Data collected using this system would allow for the creation of data driven models to investigate variations in mobility following medication intake, thus providing greater insights for optimised medication regimens in PwP.

This study aims to collect real-world data using a novel DHTS (smartphone, smartwatch, and inertial measurement unit) to assess self-reported medication adherence, quantify DMOs, and model and predict their response to medication. Within this study, specific objectives include: (i) clinically characterise our participants with PD (e.g., Hoehn and Yahr stage, disease duration etc.); (ii) collect real-world data with a connected DHTS in PwP; (iii) assess real-world, self-reported medication adherence in PwP by recording medication intakes (smartwatch reminders, time of logged intakes and acknowledged medication intake

For peer review only - http://bmjopen.bmj.com/site/about/guidelines.xhtml

events); (iv) quantify real-world DMOs (e.g., walking speed) and (v) model and predict response of DMOs to PD medication.

METHODS AND ANALYSIS

The SPIRIT reporting guidelines have been followed within this manuscript (27) (supplementary material 1). This protocol represents protocol version 2.4, April 2022.

Study design and setting

This cross-sectional, observational study will recruit 55 participants with Parkinson's disease (IRAS Number: 295771). It will be conducted at the Clinical Ageing Research Unit, Campus for Ageing and Vitality, Newcastle University NE4 5PL, Newcastle upon Tyne, with a remote home and community-based component. The study is co-funded by the Medical Research Council (MRC) Newcastle University, the United Kingdom Research Innovation, Engineering and Physical Sciences Research Council (UKRI ESPRC) and the National Institute of Health Research (NIHR). This study is a sub-study of the 'Mobilise-D – Clinical Validation Study' (IRAS Number: 289543 and REC reference: 20/PR/0792), a longitudinal study, which aims to validate a novel digital method for remote monitoring of mobility (28).

Study procedure and flow

Recruitment and screening

Participants will be identified and recruited between June 2021 – June 2024 through the "Mobilise-D - Clinical Validation Study" Newcastle upon Tyne site, and independently through local movement disorder clinics and research registries. Interested and potentially eligible participants will attend a screening appointment during which informed consent will be obtained, and eligibility confirmed. Informed consent will be taken by the Chief Investigator or a delegated member of the research team. At the beginning of the screening appointment and prior to further discussion, the individual obtaining consent will confirm that the potential participant has read the Participant Information Sheet (PIS), and will answer any questions. Upon agreement to participate in the study, the participant will sign and date the informed consent form and the researcher will witness this, and sign and date the same form (supplementary material 2 and 3). The process will then be recorded in the participants' medical records, with the original filed on site and a copy given to the participant. Those recruited through the Mobilise-D study will attend a screening appointment within the Mobilise-D first visit and those recruited independently will attend a separate screening appointment. A full outline of the recruitment procedure is detailed in Figure 1. All participants will be reimbursed for travel expenses. Due to the paucity of research exploring the influence of medication on real-world mobility in PwP using a DHTS, there is insufficient data to inform a reliable power calculation. For this study, a sample size of 55 was defined according to COnsensus-based Standards for selection of health Measurement Instruments (COSMIN) guidelines (29).

Please insert Figure 1 here.

Figure 1 caption: 'Figure 1. Flow chart of recruitment and screening procedure.'

Participants and eligibility criteria

Participants with mild to moderate disease severity will be recruited (Hoehn and Yahr stage Ⅰ-Ⅲ). The eligibility criteria (inclusion and exclusion) are in agreement with the Mobilise-D study and are detailed in Table 1. Potential participants will not be recruited if they have received and/or are due to receive a medication review within four weeks of the study assessment. Individuals with more advanced PD (Hoehn and Yahr stage IV and V) often possess a decreased ability to efficiently perform activities of daily living and experience cognitive impairment. These factors may induce difficulties to monitor

For peer review only - http://bmjopen.bmj.com/site/about/guidelines.xhtml

mobility and alter the participant's ability to interact with the DHTS, respectively. Consequently, only individuals at Hoehn and Yahr stages I-III will be recruited in this study.

Table 1. Inclusion and Exclusion Criteria.

Inclusion criteria	Exclusion criteria
• Adults aged 18 or over	• Occurrence of any of the following within 3 months prior to informed consent: myocardial infarction, hospitalisation for unstable angina, stroke, coronary artery bypass graft, percutaneous coronary intervention, implantation of a cardiac resynchronization therapy device, active treatment for cancer or other malignant disease, uncontrolled congestive heart disease (NYHA class >3), acute psychosis, major psychiatric disorders, or continued substance abuse
• Ability to consent and comply with study specific procedures	• History consistent with Dementia with Lewy Bodies, atypical parkinsonian syndromes (including multiple system atrophy or progressive supranuclear palsy, diagnosed according to accepted criteria)
• Able to read and write in English	• Repeated strokes or stepwise progression of symptoms, leading to a diagnosis of 'vascular parkinsonism'
• Patients with the clinical diagnosis of PD according to the Movement Disorder Society criteria (30)	• Drug-induced Parkinsonism
• Hoehn and Yahr stage I – III (31)	
• On stable PD medication doses (taking the same medication for four weeks or more)	
• Able to walk 4 metres independently with or without walking aids	
• Willingness to wear and interact with a smartwatch and IMU, and carry a smartphone	

Study assessments

At the beginning of the study, all participants will complete a clinic-based assessment including a range of general, clinical, PD-specific, and physical assessments (Table 2). These assessments will be performed during the first Mobilise-D visit or within 14 days of the screening appointment for those recruited independently. Following assessment completion, participants will be equipped with a novel DHTS to wear and interact with over seven consecutive days and will complete a motor complications diary (ON-OFF-Dyskinesia) each day. Participants will be free to withdraw from the study at any time, without providing reasoning and they will be given a contact point to obtain further study information.

For peer review only - http://bmjopen.bmj.com/site/about/guidelines.xhtml

Researchers who collect data pertaining to outcome measures will receive appropriate training before data collection (e.g., Good Clinical Practice training, informed consent in clinical research training, MDS-UPDRS and Montreal Cognitive Assessment training). To promote participant recruitment and retention, participants will be given detailed oral instructions, and written instructions via a PIS. The PIS will include details for day-to-day use of the devices (i.e., how to charge the devices, how to respond to medication notifications etc.) and contact details for the research team should any issues or questions arise.

Table 2. Outcome measures.

**General measures**
• Descriptive measures (year of birth, sex, living arrangement, employment status, years in education, native language, marital status)
• Anthropometric measures (height, mass, shoe size, and leg length)
• Health status (current medication and use of mobility aids (e.g., walking frame))
**Clinical measures**
• Disease Duration
• Late-Life Functional Disability Instrument (LLFDI) (32)
• Levodopa equivalent daily dose (LEDD) (33, 34)
• Frailty Phenotype (35)
**Parkinson's disease specific measures**
• Movement Disorder Society – Unified Parkinson's Disease Rating Scale (MDS-UPDRS) (30)
• Hoehn and Yahr Stages (31)
• New Freezing of Gait Questionnaire (NFOG-Q) (36)
• Montreal Cognitive Assessment (MoCA) (37)
**Physical Measures**
• Short Physical Performance Battery (SPPB) (38)
• Seven-day remote monitoring period (interact with DHTS)
**Diary, questionnaire, and qualitative questions**
• Motor complications diary (ON-OFF-dyskinesia)
• Usability questionnaire (39)
• Open-text questions

Seven-day remote monitoring period

Primary outcome measures include self-reported medication adherence (i.e. frequency, intake time, and dosage of medication) and walking speed, captured and quantified via the smartwatch and IMU, respectively. Secondary outcome measures include macro outcomes (related to volume (e.g. step count), pattern (e.g. walking bout length), and variability (e.g. walking bout length variability) of walking activity and micro outcomes (related to pace (e.g., gait speed), rhythm (e.g., step time), variability (e.g., step time variability), asymmetry (e.g., step time asymmetry), and postural control (e.g. step length asymmetry)) (40-43). DMOs obtained during the seven-day monitoring period. In addition, contextual factors captured

For peer review only - http://bmjopen.bmj.com/site/about/guidelines.xhtml

on the smartphone, including geolocation (probability of being indoors/outdoors), number of steps outside the home, stay points, common paths, and weather conditions (for more detail, see Smartphone section below).

Measurement tools and data collection

To collect data on self-reported medication adherence, DMOs, and contextual insights, participants will interact with the DHTS (smartphone, smartwatch, and IMU) over seven consecutive days. This monitoring period will commence following the first Mobilise-D visit or following the initial assessment for those recruited independently.

Smartphone to collect contextual data

As this study will be carried out remotely in the participant's home and their community, contextual factors such as weather conditions may influence DMOs. To quantify the effect of these confounding contextual factors on DMOs, participants will be advised to carry a smartphone (Samsung Galaxy S9, S10 or S21) whenever outside their house, making sure it is switched on at all times and fully charged. Participants will be provided with a charger and will be advised to charge the smartphone every night throughout the monitoring period.

The custom-made Aeqora application extension will be pre-installed onto each smartphone (Department of Computer Science, The University of Sheffield, UK). This application will utilise the phone's sensors to capture contextual information, such as, the number of steps taken outside the home per day, geolocation, stay points, common paths, and weather conditions. The application has been developed as an Android mobile application. It is made up of three components: (i) the core tracker, (ii) the interface, and (iii) the server infrastructure, which collects data across users (44). The core tracker has been adapted from a library developed by the University of Sheffield (45). This tracker identifies the type of activity (e.g., walking) from the phone's internal sensors (accelerometer and gyroscope). It continually operates in the background to sense mobility features using these sensors and location services, such as Bluetooth (44). To identify the weather conditions at the time of measurement, the closest weather station to the participant's location will be utilised. Raw sensor data will be collected and stored in real-time onto a local database which is synchronised with a remote database when the phone is recharging. No participant identifiable information will be sent to the server. The data is synched to the remote database via a Secure Socket Layer (SSL) and Transports Layer Security (TLS) 3.0 protocol. To ensure privacy and security when communicating sensitive data an SSL/TSL certificate is exchanged between client and server, which establishes the identity and trust between them.

Smartwatch to assess real-world, self-reported medication adherence

To assess real-world, self-reported medication adherence, participants will wear and interact with a smartwatch (TicWatch Pro 3, Mobvoi) throughout the monitoring period. During the clinic visit, a researcher will manually input the participant's medication reminders onto the Aeqora application. This application will then send notifications from the smartphone to the smartwatch to remind participants of their PD-specific medication intake times and allow them to log the times at which their medication was taken, clicking either "YES" or "NO" on the watch face when prompted (Figure 2). The watch will also allow participants to log any additional PD-specific medication they have taken ('as required'). Participants will be provided with a smartwatch charger and will be required to remove and charge the smartwatch overnight throughout the monitoring period.

IMU to quantify digital mobility outcomes

To quantify primary and secondary DMOs, participants will continuously wear an IMU (Axivity AX6, Axivity Ltd, Newcastle upon Tyne, UK, including triaxial accelerometer and gyroscope, dimensions 23 x

For peer review only - http://bmjopen.bmj.com/site/about/guidelines.xhtml

32.5 x 8.9mm, mass 11g, frequency 100Hz, gyroscope range ± 2000 degrees per second and accelerometer range ± 8g) throughout the monitoring period. The IMU will be secured to the participant's lower back, at the fifth lumbar vertebrae (44). It will be affixed using a hydrogel/ hydrocolloid adhesive and a hypoallergenic plaster patch. Participants will be advised to continue with their usual activities and not change their routine. Throughout the monitoring period, all IMU data will be stored locally on the IMU hardware. After the monitoring period, participants will return the IMUs via pre-paid evelopes and data will be downloaded and uploaded onto the eScience (e-SC) (a cloud based storage platform) CiC Data Management platform (46, 47) (see data management section). The IMU will be worn at all times, including during the night and although the IMU is waterproof participants will be advised to remove it during prolonged water exposure (e.g., swimming, taking a bath).

Please insert Figure 2 here.

Figure 2 caption: 'Figure 2. Digital Health Technology System (DHTS). (A) Inertial Measurement Unit (IMU), which monitors mobility. (B) Smartwatch which assesses self-reported medication adherence, by notifying participants when to take their medication and allowing them to log the time at which they took their medication. (C) Smartphone which collects contextual data.'

Diary to monitor motor complications

During the seven-day monitoring period, participants will fill in a motor complications diary (paper-based) for each day, indicating their "OFF-status" when they feel their medication is not working and dyskinesias, when they experience involuntary movements. The diary will record data over 16 hours each day from 6:00am to 22:00pm, from days one to seven (supplementary material 4).

Assessment of participants' experience with the DHTS

Questionnaire to evaluate usability of the DHTS

To evaluate the participants' experience with the DHTS, participants will complete an adapted version of Rabinovich et al's 12-item usability questionnaire at the end of the monitoring period (supplementary material 5) (39). This questionnaire investigates usability on a 5-point ordinal scale, from 5 (most favourable) to 1 (least favourable), and 'no opinion' scored as 3. The questions identify participant's attitude towards the DHTS, its ease of use and comfort, such as, "I experienced technical problems" and "The monitors interfered with my normal activity". Participants will also rate the DHTS on a scale from 0 (worst score) to 100 (best score).

Qualitative feedback on the DHTS and its individual devices will be collected via open-text questions added to the end of the questionnaire (supplementary material 5). These questions require participants to "give any other comments on the DHTS and its components", to describe the "problems" they experienced when using the DHTS and its components, as well as any features they liked about the system and its individual devices. All devices will be returned at the end of the assessment period, in addition to the questionnaire and motor complications diary, using pre-paid tracked envelopes.

Feasibility of DHTS and motor complications diary

To assess whether the DHTS and motor complications diary are feasible for everyday use in PwP, this study will assess their feasibility (see data analysis section for details).

Patient and Public Involvement

As this study is a sub-study of the "Mobilise-D – Clinical Validation Study" insights from patient and public advisory groups which informed the Mobilise-D study were used to inform the development of this study. The Mobilise-D Study Participant Information Sheet (PIS) was reviewed and modified by relevant patient groups and was used as a guide for this study's PIS. Participants in this study will provide

For peer review only - http://bmjopen.bmj.com/site/about/guidelines.xhtml

feedback on usability of the DHTS, which will inform future studies. Study insights will be fed back to patient stakeholder groups within the Mobilise-D consortium.

Data and Statistical Analysis

Data analysis

To quantify DMOs, mobility data from the IMUs will be analysed using validated Matlab® algorithms (R2019a, Mathworks, California, United States) (47, 48). To investigate the effect of medication on DMOs, medication intake timestamps (captured via the smartwatch) and mobility data from the IMU will be utilised. The effect of contextual factors (e.g., weather conditions and probability of being indoors/outdoors) on DMOs will be considered when interpreting the medication and mobility data.

Responses to open-text questions will be analysed using a hybrid approach, with both deductive and inductive methods used to analyse responses. Responses will be grouped into three themes: feasibility, usability, and recommendations for improvement. Once grouped, key sub-themes within each group (i.e., ease of use for the usability theme) will be identified following the same approach.

To assess the feasibility of the DHTS devices and the motor complications diary, we will assess whether the intended data was collected; specifically contextual data, self-reported medication adherence data, and mobility data for the smartphone, smartwatch, and IMU, respectively; and motor complications data for the motor complications diary (49). A summary of feasibility measures and their outcomes is included in Table 3.

Table 3. Summary of feasibility and outcomes extracted from DHTS and motor complications diary.

Device	Measure of feasibility	Outcomes extracted
**Smartphone**	Percentage of datasets collected over seven days and percentage of days missing	Number of steps taken outside the participant's home, per day
**Smartwatch**	Percentage of participants interacting with the smartwatch over seven day monitoring period	Number of interactions recorded on smartwatch
**IMU**	Percentage of datasets collected over seven day monitoring period	Walking speed and number of steps per day
**Motor complications diary**	Percentage of diaries returned and legible	Time spent in an ON/OFF state or experiencing dyskinesia

Only participants whose mobility data is collected for at least 3 days, will be included in analysis. If participants do not complete one of the study assessments (e.g., SPPB) or their data from the devices is not collected, their remaining data will still be included in analysis. Assuming missing data is at random, modern statistical techniques will be utilised to handle missing data and will be compared with a complete case approach (50).

For peer review only - http://bmjopen.bmj.com/site/about/guidelines.xhtml

Statistical analysis

Participants' descriptive, clinical, and PD specific characteristics, as well as data derived from the DHTS and motor complications diary data, will be analysed using descriptive statistics, including percentage and frequency, mean and standard deviation or median and interquartile range, as appropriate.

Functional data analysis and deep learning techniques (51) will be used to model and predict DMOs response to medication, and potential effects of contextual factors. DMO trends will be analysed between medication intake times and wearing-off effects will be evaluated via a customised scoring system. This scoring system will identify the wear off periods, grouping them relative to the strength of their effect. Nonlinear time series analysis will assess changes in DMOs between medication intakes (48, 52). Data collected using motor complications diaries will be used to optimise time series modelling of DMOs.

Should the data collected require an adjusted methodology, proposed statistical methods will be amended following completion of data collection.

Data management

All aspects of data management will adhere to the FAIR guiding principles, ensuring that data are findable, accessible, interoperable, and reusable (53), as well as being attributable, legible, contemporaneous, original, and accurate (ALCOA) (54). The CiC data management platform has been created on the open source, cloud-based platform e-Science Central (e-SC) (46, 47). This platform securely stores and shares data and has data processing capabilities.

This is a low risk, observational study which does not require a Data Monitoring Committee. The Data Management Group (SMG) will be responsible for managing data sharing requests, and upon data requests will meet with external researchers. This protocol received input from PD and clinical experts (LR, AY); technical experts (SDD, LA, JSQ) gave their input on data collection methods, outcomes, and eligibility criteria.

Data collection, storage, and archiving

To maintain confidentiality, participant data will be collected in a coded, anonymised manner using unique identification codes. Data will be directly uploaded onto the e-SC CiC platform or via third-party platforms (e.g., weather data). All researchers will be trained to correctly enter and upload data to relevant platforms. All data collected on paper case report forms will be signed, dated, scanned, and uploaded onto a secure network drive with restricted access. The e-SC CiC platform will be implemented using secure Amazon Web Services located within the European Union. The clinical site will keep original records of all paper forms in secure conditions until the study is completed. Long term data storage will be in accordance with the Newcastle Joint Office policy for archiving, with data security arrangements made in accordance with Newcastle upon Tyne NHS Foundation Trust policies. The clinical site will be responsible for archiving study documents for a period which is in keeping with the institutional or national guidelines pertaining to the site. Table 4 summarises the data transfer pipeline.

For peer review only - http://bmjopen.bmj.com/site/about/guidelines.xhtml

Table 4. Summary of standardised and secure data transfer pipeline.

Data type	Data pipeline
**Clinical and Demographic data**	Data will be collected on paper forms, which will be scanned and uploaded to a secured password protected University drive. Processed data will be entered onto the database. Paper forms will be stored in the Gait Laboratory at the Clinical Ageing Research Unit, with access restricted to the laboratory research team.
**Mobility data from IMU**	When using the Axivity AX6 sensor, raw data will be directly uploaded to the data management platform on e-SC from the clinical site.
**Self-reported medication adherence data (smartwatch and smartphone)**	Data that are logged on the smartphone will be transferred to the University of Sheffield (USFD) servers. Processed data (e.g., acknowledged medication intake times) will then be transferred from USFD servers and uploaded to eSC platform via a secured system (e.g. API or web portal).
**Contextual data from smartphone**	Data will be transferred from the smartphone to the USFD servers. Processed data (annotations) will be transferred from USFD servers and uploaded to the eSC platform via a secured system (e.g. API or web portal).
**Usability questionnaire, open-text questions, and motor complications diary**	Usability questionnaires, responses to open-text questions, and diary data will be collected with paper forms. These forms will be scanned and uploaded to a secured password protected University drive. Processed data will be entered onto a database.

Ethics and dissemination

This single centre study is sponsored by the Newcastle upon Tyne Hospitals NHS Foundation Trust (NuTH) UK. Ethical approval was obtained from the London - 142 Westminster Research Ethics Committee (REC reference: 21/PR/0469). The study will be conducted in accordance with Good Clinical Practice standards, the declaration of Helsinki, and The European Code of Conduct for Research Integrity (55). This study will be included in the NIHR Clinical Network Portfolio and has been registered in the ISRCTN registry (ISRCTN number: 13156149). Informed consent will be obtained by a trained researcher. The NHS indemnity scheme and Newcastle University are liable for harm to participants arising from the management and conduct of the study, and from its design, respectively.

As this is an observational study, there is no adverse event reporting planned. Should any Serious Adverse Events (SAEs) which are related to the administration of the research procedure occur, the sponsor and Chief Investigator will be informed. On behalf of the sponsor, the Newcastle Joint Research Office Governance Team will be responsible for assessing protocol deviations, serious breaches, and violations, as well as reporting Serious Breaches to the REC. The Chief Investigator is responsible for reporting protocol deviations and violations. Any protocol amendments will be communicated to the sponsor with all required amendment documents completed. The sponsor will be required to agree and accept any modifications, before amendments can be submitted to the NHS REC for their approval.

For peer review only - http://bmjopen.bmj.com/site/about/guidelines.xhtml

Dissemination policy

Newsletters will be sent to participants to inform them of study outcomes. Research will be disseminated through peer reviewed scientific journals, internal reports, conference presentations, and relevant websites. We will follow the International Committee of Medical Journal Editors recommendations for authorship eligibility.

Access to data

We plan to indefinitely maintain, the complete, anonymised dataset on the e-SC platform. On study completion, this dataset will be made available to the wider research community; upon request and will be shared with approved collaborators nationally and internationally for peer review, scientifically sound studies. Participants will have the right to access a copy of their data or request its removal following an appropriate request. These rights will terminate following dataset anonymisation and after the study key code is destroyed. The lead clinical investigator has overall responsibility for study execution and can access the full dataset. Data analysts, who are responsible for implementation of data protocol, will have access to the full-anonymised dataset. Statisticians will only have access to the full-anonymised datasets and will not have access to raw data files. Access to study data will be given to authorised representatives from the Sponsor, host institution, and regulatory authorities to allow study related audits, monitoring and inspections – in line with participant consent.

Conclusion

To the authors' knowledge, this ongoing study will be pioneering in utilising this DHTS to model and predict the influence of medication on mobility in PwP. Insights from this study will enhance our understanding of the effect of medication on mobility in the real-world and may contribute to more optimised medication regimens for PwP, thereby improving their quality of life. Clinical findings gained from this protocol will generate evidence to support the use of DHTS for enhancing care and clinical management of PwP (24).

Trial status

The first phase of recruitment has finished, with data collected in 30 PwP. Recruitment for the second phase of the study will start in February 2023 and will continue until June 2024 or until the desired sample size has been reached.

Footnotes

Author contributions

Study design: SDD, LA, AY, LR. Ethical approval and clinical oversight: SDD, LA, AY, LR. Manuscript initial drafting: EP, HD. Figures and tables preparation: EP, HD. Manuscript revision: EP, HD, SDD, LA. Intellectual contribution: EP, HD, HB, FC, NI, JE, MN, JQS, AY, LR, LA, SDD. All authors have provided critical intellectual input during manuscript revision. All authors have reviewed and approved the submitted manuscript. The corresponding author attests that all listed authors meet authorship criteria and that no others meeting the criteria have been omitted.

Funding

The study is co-funded by the Medical Research Council (MRC) Newcastle University (MC_PC_19047, NU-002884), the United Kingdom Research Innovation, Engineering and Physical Sciences Research Council (UKRI EPSRC) and the National Institute of Health Research (NIHR) (Grant Ref: EP/W031590/1, "Transforming care and health at home and enabling independence"). This study was also supported by the Mobilise-D project which has received from the Innovative Medicines Initiative 2 Joint Undertaking, under the grant agreement No. 820820. This JU receives support from the European

For peer review only - http://bmjopen.bmj.com/site/about/guidelines.xhtml

Union's Horizon 2020 research and innovation program and the European Federation of Pharmaceutical Industries and Associations (EFPIA).

The research was supported by the National Institute for Health and Care Research (NIHR) Newcastle Biomedical Research Centre based at The Newcastle upon Tyne Hospitals NHS Foundation Trust, Newcastle University and the Cumbria, Northumberland and Tyne and Wear (CNTW) NHS Foundation Trust.

The research was also supported by NIHR Newcastle Clinical Research Facility (CRF) Infrastructure funding. All opinions are those of the authors, not the funders. Views expressed are those of the authors and do not necessarily reflect those of the NHS, the NIHR, UKRI EPSRC, Department of Health, the IMI, the European Union, the EFPIA or Associated Partners.

Competing interests

FC was CEO and shareholder of Aeqora Ltd. JE and MN are employees of McRoberts. SDD reports consultancy activity with Hoffmann-La Roche Ltd. outside of this study.

Acknowledgments

The authors would like to acknowledge and thank all collaborators of this study. We gratefully acknowledge the contribution of our much-missed colleague Dr Richard Dodds, who sadly passed away before this study could be completed.

References

1. Studenski S, Perera S, Patel K, et al. Gait speed and survival in older adults. Jama. 2011;305(1):50-8. doi: 10.1001/jama.2010.1923
2. Shumway-Cook A, Ciol MA, Yorkston KM, et al. Mobility Limitations in the Medicare Population: Prevalence and Sociodemographic and Clinical Correlates. Journal of the American Geriatrics Society. 2005;53(7):1217-21. doi: https://doi.org/10.1111/j.1532-5415.2005.53372.x
3. Armstrong MJ, Okun MS. Diagnosis and Treatment of Parkinson Disease: A Review. Jama. 2020;323(6):548-60. doi: 10.1001/jama.2019.22360
4. Paker N, Bugdayci D, Goksenoglu G, et al. Gait speed and related factors in Parkinson's disease. J Phys Ther Sci. 2015;27(12):3675-9. doi: 10.1589/jpts.27.3675
5. Malek N, Grosset DG. Medication adherence in patients with Parkinson's disease. CNS Drugs. 2015;29(1):47-53. doi: 10.1007/s40263-014-0220-0
6. Daley DJ, Myint PK, Gray RJ, et al. Systematic review on factors associated with medication non-adherence in Parkinson's disease. Parkinsonism Relat Disord. 2012;18(10):1053-61. doi: 10.1016/j.parkreldis.2012.09.004
7. Kulkarni AS, Balkrishnan R, Anderson RT, et al. Medication adherence and associated outcomes in medicare health maintenance organization-enrolled older adults with Parkinson's disease. Movement Disorders. 2008;23(3):359-65. doi: https://doi.org/10.1002/mds.21831
8. Campbell F, Ashburn A, Thomas P, et al. An exploratory study of the consistency of balance control and the mobility of people with Parkinson's disease (PD) between medication doses. Clin Rehabil. 2003;17(3):318-24. doi: 10.1191/0269215503cr614oa
9. Araújo-Silva F, Santinelli FB, Felipe IIL, et al. Temporal dynamics of cortical activity and postural control in response to the first levodopa dose of the day in people with Parkinson's disease. Brain Res. 2022;1775:147727. doi: 10.1016/j.brainres.2021.147727
10. Ávila de Oliveira J, Bazán PR, de Oliveira CEN, et al. The effects of levodopa in the spatiotemporal gait parameters are mediated by self-selected gait speed in Parkinson's disease. Eur J Neurosci. 2021;54(11):8020-8. doi: 10.1111/ejn.15522

For peer review only - http://bmjopen.bmj.com/site/about/guidelines.xhtml

11. Troisi Lopez E, Minino R, Sorrentino P, et al. Sensitivity to gait improvement after levodopa intake in Parkinson's disease: A comparison study among synthetic kinematic indices. PLoS One. 2022;17(5):e0268392. doi: 10.1371/journal.pone.0268392
12. Wu Z, Xu H, Zhu S, et al. Gait Analysis of Old Individuals with Mild Parkinsonian Signs and Those Individuals' Gait Performance Benefits Little from Levodopa. Risk Manag Healthc Policy. 2021;14:1109-18. doi: 10.2147/rmhp.S291669
13. Smulders K, Dale ML, Carlson-Kuhta P, et al. Pharmacological treatment in Parkinson's disease: Effects on gait. Parkinsonism & Related Disorders. 2016;31:3-13. doi: https://doi.org/10.1016/j.parkreldis.2016.07.006
14. Curtze C, Nutt JG, Carlson-Kuhta P, et al. Levodopa Is a Double-Edged Sword for Balance and Gait in People With Parkinson's Disease. Movement Disorders. 2015;30(10):1361-70. doi: https://doi.org/10.1002/mds.26269
15. Galna B, Lord S, Burn DJ, et al. Progression of gait dysfunction in incident Parkinson's disease: Impact of medication and phenotype. Movement Disorders. 2015;30(3):359-67. doi: https://doi.org/10.1002/mds.26110
16. Bryant MS, Rintala DH, Hou JG, et al. Gait variability in Parkinson's disease: influence of walking speed and dopaminergic treatment. Neurol Res. 2011;33(9):959-64. doi: 10.1179/1743132811y.0000000044
17. Liu Y, Zhang G, Tarolli CG, et al. Monitoring gait at home with radio waves in Parkinson's disease: A marker of severity, progression, and medication response. Sci Transl Med. 2022;14(663):eadc9669. doi: 10.1126/scitranslmed.adc9669
18. Atrsaei A, Corrà MF, Dadashi F, et al. Gait speed in clinical and daily living assessments in Parkinson's disease patients: performance versus capacity. NPJ Parkinsons Dis. 2021;7(1):24. doi: 10.1038/s41531-021-00171-0
19. Del Din S, Galna B, Godfrey A, et al. Analysis of Free-Living Gait in Older Adults With and Without Parkinson's Disease and With and Without a History of Falls: Identifying Generic and Disease-Specific Characteristics. J Gerontol A Biol Sci Med Sci. 2019;74(4):500-6. doi: 10.1093/gerona/glx254
20. Del Din S, Godfrey A, Rochester L. Validation of an Accelerometer to Quantify a Comprehensive Battery of Gait Characteristics in Healthy Older Adults and Parkinson's Disease: Toward Clinical and at Home Use. IEEE J Biomed Health Inform. 2016;20(3):838-47. doi: 10.1109/jbhi.2015.2419317
21. Zhang T, Dong L, Jing H, et al. Smartphone Applications in the Management of Parkinson's Disease in a Family Setting: An Opinion Article. Frontiers in Neurology. 2021;12. doi: 10.3389/fneur.2021.668953
22. Roth N, Wieland GP, Kuderle A, et al. Do We Walk Differently at Home? A Context-Aware Gait Analysis System in Continuous Real-World Environments. Annu Int Conf IEEE Eng Med Biol Soc. 2021;2021:1932-5. doi: 10.1109/embc46164.2021.9630378
23. Fritz S, Lusardi M. White paper: "walking speed: the sixth vital sign". J Geriatr Phys Ther. 2009;32(2):46-9. doi, 20039582
24. Dorsey ER, Bloem BR, Okun MS. A New Day: The Role of Telemedicine in Reshaping Care for Persons With Movement Disorders. Mov Disord. 2020;35(11):1897-902. doi: 10.1002/mds.28296
25. Joshi R, Bronstein JM, Keener A, et al. PKG Movement Recording System Use Shows Promise in Routine Clinical Care of Patients With Parkinson's Disease. Frontiers in Neurology. 2019;10. doi: 10.3389/fneur.2019.01027
26. Chen L, Cai G, Weng H, et al. More Sensitive Identification for Bradykinesia Compared to Tremors in Parkinson's Disease Based on Parkinson's KinetiGraph (PKG). Front Aging Neurosci. 2020;12:594701. doi: 10.3389/fnagi.2020.594701
27. Chan AW, Tetzlaff JM, Gøtzsche PC, et al. SPIRIT 2013 explanation and elaboration: guidance for protocols of clinical trials. Bmj. 2013;346:e7586. doi: 10.1136/bmj.e7586

For peer review only - http://bmjopen.bmj.com/site/about/guidelines.xhtml

28. Mikolaizak AS, Rochester L, Maetzler W, et al. Connecting real-world digital mobility assessment to clinical outcomes for regulatory and clinical endorsement-the Mobilise-D study protocol. PLoS One. 2022;17(10):e0269615. doi: 10.1371/journal.pone.0269615
29. COSMIN. COnsensus-based Standards for the selection of health Measurement INstruments. [Available from: https://www.cosmin.nl/.
30. Goetz CG, Tilley BC, Shaftman SR, et al. Movement Disorder Society-sponsored revision of the Unified Parkinson's Disease Rating Scale (MDS-UPDRS): scale presentation and clinimetric testing results. Mov Disord. 2008;23(15):2129-70. doi: 10.1002/mds.22340
31. Hoehn MM, Yahr MD. Parkinsonism: onset, progression and mortality. Neurology. 1967;17(5):427-42. doi: 10.1212/wnl.17.5.427
32. Jette AM, Haley SM, Coster WJ, et al. Late life function and disability instrument: I. Development and evaluation of the disability component. J Gerontol A Biol Sci Med Sci. 2002;57(4):M209-16. doi: 10.1093/gerona/57.4.m209
33. Tomlinson CL, Stowe R, Patel S, et al. Systematic review of levodopa dose equivalency reporting in Parkinson's disease. Mov Disord. 2010;25(15):2649-53. doi: 10.1002/mds.23429
34. Schade S, Mollenhauer B, Trenkwalder C. Levodopa Equivalent Dose Conversion Factors: An Updated Proposal Including Opicapone and Safinamide. Mov Disord Clin Pract. 2020;7(3):343-5. doi: 10.1002/mdc3.12921
35. Fried LP, Tangen CM, Walston J, et al. Frailty in Older Adults: Evidence for a Phenotype. The Journals of Gerontology: Series A. 2001;56(3):M146-M57. doi: 10.1093/gerona/56.3.M146
36. Nieuwboer A, Rochester L, Herman T, et al. Reliability of the new freezing of gait questionnaire: agreement between patients with Parkinson's disease and their carers. Gait Posture. 2009;30(4):459-63. doi: 10.1016/j.gaitpost.2009.07.108
37. Nasreddine ZS, Phillips NA, Bédirian V, et al. The Montreal Cognitive Assessment, MoCA: a brief screening tool for mild cognitive impairment. J Am Geriatr Soc. 2005;53(4):695-9. doi: 10.1111/j.1532-5415.2005.53221.x
38. Guralnik JM, Simonsick EM, Ferrucci L, et al. A Short Physical Performance Battery Assessing Lower Extremity Function: Association With Self-Reported Disability and Prediction of Mortality and Nursing Home Admission. Journal of Gerontology. 1994;49(2):M85-M94. doi: 10.1093/geronj/49.2.M85
39. Rabinovich RA, Louvaris Z, Raste Y, et al. Validity of physical activity monitors during daily life in patients with COPD. European Respiratory Journal. 2013;42(5):1205-15. doi: 10.1183/09031936.00134312
40. Lord S, Galna B, Rochester L. Moving forward on gait measurement: Toward a more refined approach. Movement Disorders. 2013;28(11):1534-43. doi: https://doi.org/10.1002/mds.25545
41. Del Din S, Godfrey A, Galna B, et al. Free-living gait characteristics in ageing and Parkinson's disease: impact of environment and ambulatory bout length. Journal of NeuroEngineering and Rehabilitation. 2016;13(1):46. doi: 10.1186/s12984-016-0154-5
42. Hickey A, Del Din S, Rochester L, et al. Detecting free-living steps and walking bouts: validating an algorithm for macro gait analysis. Physiol Meas. 2017;38(1):N1-n15. doi: 10.1088/1361-6579/38/1/n1
43. Morris R, Hickey A, Del Din S, et al. A model of free-living gait: A factor analysis in Parkinson's disease. Gait Posture. 2017;52:68-71. doi: 10.1016/j.gaitpost.2016.11.024
44. Mazzà C, Alcock L, Aminian K, et al. Technical validation of real-world monitoring of gait: a multicentric observational study. BMJ Open. 2021;11(12):e050785. doi: 10.1136/bmjopen-2021-050785
45. Ciravegna F, Gao J, Ireson N, et al. Active 10: Brisk Walking to Support Regular Physical Activity. Proceedings of the 13th EAI International Conference on Pervasive Computing Technologies for Healthcare; Trento, Italy: Association for Computing Machinery; 2019. p. 11–20.
46. Hiden H, Woodman S, Watson P, et al. Developing cloud applications using the e-Science Central platform. Philos Trans A Math Phys Eng Sci. 2013;371(1983):20120085. doi: 10.1098/rsta.2012.0085

For peer review only - http://bmjopen.bmj.com/site/about/guidelines.xhtml

47. Din SD, Hickey A, Woodman S, et al. Accelerometer-based gait assessment: Pragmatic deployment on an international scale. 2016 IEEE Statistical Signal Processing Workshop (SSP). 2016:1-5. doi: 10.1109/SSP.2016.7551794
48. Rehman RZU, Buckley C, Mico-Amigo ME, et al. Accelerometry-Based Digital Gait Characteristics for Classification of Parkinson's Disease: What Counts? IEEE Open J Eng Med Biol. 2020;1:65-73. doi: 10.1109/ojemb.2020.2966295
49. World Health Organization. Monitoring and Evaluating Digital Health Interventions: A Practical Guide to Conducting Research and Assessment.; 2016.
50. Kang H. The prevention and handling of the missing data. Korean J Anesthesiol. 2013;64(5):402-6. doi: 10.4097/kjae.2013.64.5.402
51. Shi JQ, T C. Gaussian process regression analysis for functional data: CRC Press; 2011.
52. Del Din S, Godfrey A, Mazzà C, et al. Free-living monitoring of Parkinson's disease: Lessons from the field. Mov Disord. 2016;31(9):1293-313. doi: 10.1002/mds.26718
53. The European Commission D-GfRI. H2020 Programme Guidelines on FAIR Data. [Available from: https://ec.europa.eu/research/participants/docs/h2020-funding-guide/cross-cutting-issues/open-access-data-management/data-management_en.htm.
54. Management in Horizon 2020. 2016 [Available from: https://ec.europa.eu/research/participants/data/ref/h2020/grants_manual/hi/oa_pilot/h2020-hi-oa-data-mgt_en.pdf.
55. Association WM. World Medical Association Declaration of Helsinki: Ethical Principles for Medical Research Involving Human Subjects. JAMA. 2013;310(20):2191-4. doi: 10.1001/jama.2013.281053

For peer review only - http://bmjopen.bmj.com/site/about/guidelines.xhtml

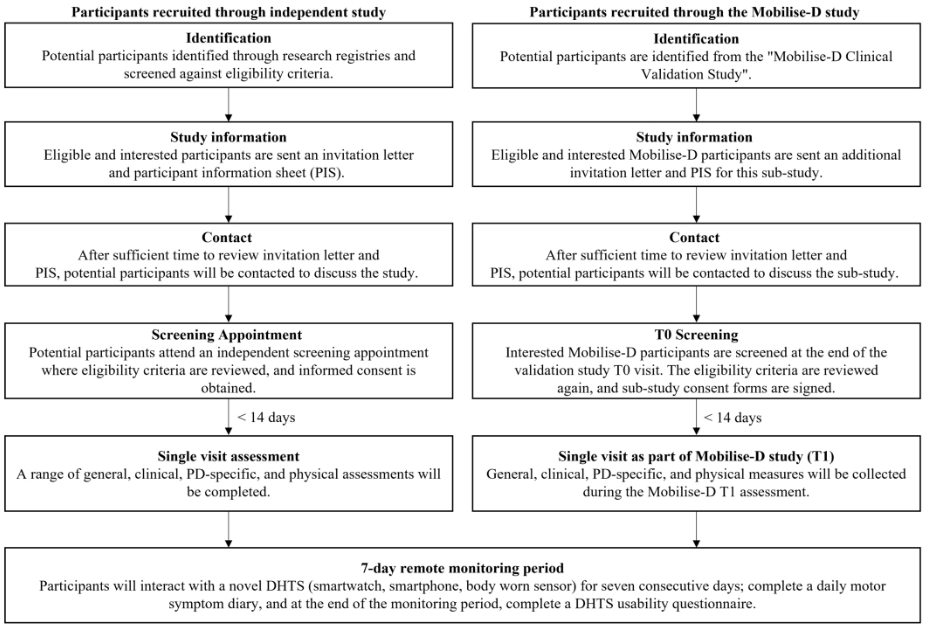

Figure 1. Flow chart of recruitment and screening procedure.

285x185mm (300 x 300 DPI)

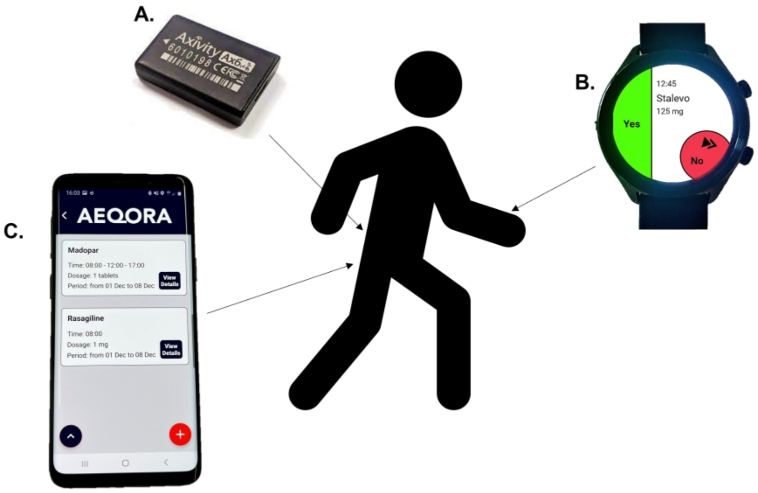

Figure 2 caption: ‘Figure 2. Digital Health Technology System (DHTS). (A) Inertial Measurement Unit (IMU), which monitors mobility. (B) Smartwatch which assesses self-reported medication adherence, by notifying participants when to take their medication and allowing them to log the time at which they took their medication. (C) Smartphone which collects contextual data.

306x218mm (300 x 300 DPI)

SPIRIT | CONSORT OUTCOMES Reporting Guidelines for Trial Outcomes

SPIRIT-Outcomes 2022 Checklist (for combined completion of SPIRIT 2013 and SPIRIT-Outcomes 2022 items)[a]

Section	Item No.	SPIRIT 2013 Item	SPIRIT-Outcomes 2022 item	Location Reported[b]
**Administrative information**				
Title	1	Descriptive title identifying the study design, population, interventions, and, if applicable, trial acronym	-	
Trial registration	2a	Trial identifier and registry name. If not yet registered, name of intended registry	-	
	2b	All items from the World Health Organization Trial Registration Data Set	-	
Protocol version	3	Date and version identifier	-	
Funding	4	Sources and types of financial, material, and other support	-	
Roles and responsibilities	5a	Names, affiliations, and roles of protocol contributors	-	
	5b	Name and contact information for the trial sponsor	-	
	5c	Role of study sponsor and funders, if any, in study design; collection, management, analysis, and interpretation of data; writing of the report; and the decision to submit the report for publication, including whether they will have ultimate authority over any of these activities	-	
	5d	Composition, roles, and responsibilities of the coordinating centre, steering committee, endpoint adjudication committee, data management team, and other individuals or groups overseeing the trial, if applicable (see Item 21a for data monitoring committee)	-	
**Introduction**				
Background and rationale	6a	Description of research question and justification for undertaking the trial, including summary of relevant studies (published and unpublished) examining benefits and harms for each intervention	-	
	6b	Explanation for choice of comparators	-	
Objectives	7	Specific objectives or hypotheses	-	

For peer review only

For peer review only - http://bmjopen.bmj.com/site/about/guidelines.xhtml

Section	Item No.	SPIRIT 2013 Item	SPIRIT-Outcomes 2022 item	Location Reported[b]
Trial design	8	Description of trial design including type of trial (eg, parallel group, crossover, factorial, single group), allocation ratio, and framework (eg, superiority, equivalence, noninferiority, exploratory)	-	
**Methods: Participants, interventions, and outcomes**				
Study setting	9	Description of study settings (eg, community clinic, academic hospital) and list of countries where data will be collected. Reference to where list of study sites can be obtained	-	
Eligibility criteria	10	Inclusion and exclusion criteria for participants. If applicable, eligibility criteria for study centres and individuals who will perform the interventions (eg, surgeons, psychotherapists)	-	
Interventions	11a	Interventions for each group with sufficient detail to allow replication, including how and when they will be administered (for specific guidance see TIDieR checklist and guide)	-	
	11b	Criteria for discontinuing or modifying allocated interventions for a given trial participant (eg, drug dose change in response to harms, participant request, or improving/worsening disease)	-	
	11c	Strategies to improve adherence to intervention protocols, and any procedures for monitoring adherence (eg, drug tablet return, laboratory tests)	-	
	11d	Relevant concomitant care and interventions that are permitted or prohibited during the trial	-	
Outcomes	12	Primary, secondary, and other outcomes, including the specific measurement variable (eg, systolic blood pressure), analysis metric (eg, change from baseline, final value, time to event), method of aggregation (eg, median, proportion), and time point for each outcome. Explanation of the clinical relevance of chosen efficacy and harm outcomes is strongly recommended	-	

For peer review only - http://bmjopen.bmj.com/site/about/guidelines.xhtml

Section	Item No.	SPIRIT 2013 Item	SPIRIT-Outcomes 2022 item	Location Reported[b]
	12.1		Provide a rationale for the selection of the domain for the trial's primary outcome	
	12.2		If the analysis metric for the primary outcome represents within-participant change, define and justify the minimal important change in individuals	
	12.3		If the outcome data collected are continuous but will be analyzed as categorical (method of aggregation), specify the cutoff values to be used	
	12.4		If outcome assessments will be performed at several time points after randomization, state the time points that will be used for analysis	
	12.5		If a composite outcome is used, define all individual components of the composite outcome	
Participant timeline	13	Time schedule of enrolment, interventions (including any run-ins and washouts), assessments, and visits for participants. A schematic diagram is highly recommended (see Figure)	-	
Sample size	14	Estimated number of participants needed to achieve study objectives and how it was determined, including clinical and statistical assumptions supporting any sample size calculations	-	
	14.1		Define and justify the target difference between treatment groups (eg, the minimal important difference)	
Recruitment	15	Strategies for achieving adequate participant enrolment to reach target sample size	-	
**Methods: Assignment of interventions (for controlled trials)**				
Allocation:				
Sequence generation	16a	Method of generating the allocation sequence (eg, computer-generated random numbers), and list of any factors for stratification. To reduce predictability of a random sequence, details of any planned restriction (eg, blocking) should be provided in a separate document that is unavailable to those who enrol participants or assign interventions	-	

For peer review only - http://bmjopen.bmj.com/site/about/guidelines.xhtml

Section	Item No.	SPIRIT 2013 Item	SPIRIT-Outcomes 2022 item	Location Reported[b]
Allocation concealment mechanism	16b	Mechanism of implementing the allocation sequence (eg, central telephone; sequentially numbered, opaque, sealed envelopes), describing any steps to conceal the sequence until interventions are assigned	-	
Implementation	16c	Who will generate the allocation sequence, who will enrol participants, and who will assign participants to interventions	-	
Blinding (masking)	17a	Who will be blinded after assignment to interventions (eg, trial participants, care providers, outcome assessors, data analysts), and how	-	
	17b	If blinded, circumstances under which unblinding is permissible, and procedure for revealing a participant's allocated intervention during the trial	-	
**Methods: Data collection, management, and analysis**				
Data collection methods	18a	Plans for assessment and collection of outcome, baseline, and other trial data, including any related processes to promote data quality (eg, duplicate measurements, training of assessors) and a description of study instruments (eg, questionnaires, laboratory tests) along with their reliability and validity, if known. Reference to where data collection forms can be found, if not in the protocol	-	
	18a.1		Describe what is known about the responsiveness of the study instruments in a population similar to the study sample	
	18a.2		Describe who will assess the outcome (eg, nurse, parent)	
	18b	Plans to promote participant retention and complete follow-up, including list of any outcome data to be collected for participants who discontinue or deviate from intervention protocols	-	

For peer review only - http://bmjopen.bmj.com/site/about/guidelines.xhtml

SPIRIT | CONSORT OUTCOMES
Reporting Guidelines for Trial Outcomes

Section	Item No.	SPIRIT 2013 Item	SPIRIT-Outcomes 2022 item	Location Reported[b]
Data management	19	Plans for data entry, coding, security, and storage, including any related processes to promote data quality (eg, double data entry; range checks for data values). Reference to where details of data management procedures can be found, if not in the protocol	-	
Statistical methods	20a	Statistical methods for analysing primary and secondary outcomes. Reference to where other details of the statistical analysis plan can be found, if not in the protocol	-	
	20a.1		Describe any planned methods to account for multiplicity in the analysis or interpretation of the primary and secondary outcomes (eg, coprimary outcomes, same outcome assessed at multiple time points, or subgroup analyses of an outcome)	
	20b	Methods for any additional analyses (eg, subgroup and adjusted analyses)	-	
	20c	Definition of analysis population relating to protocol non-adherence (eg, as randomised analysis), and any statistical methods to handle missing data (eg, multiple imputation)	-	
**Methods: Monitoring**				
Data monitoring	21a	Composition of data monitoring committee (DMC); summary of its role and reporting structure; statement of whether it is independent from the sponsor and competing interests; and reference to where further details about its charter can be found, if not in the protocol. Alternatively, an explanation of why a DMC is not needed	-	
	21b	Description of any interim analyses and stopping guidelines, including who will have access to these interim results and make the final decision to terminate the trial	-	
Harms	22	Plans for collecting, assessing, reporting, and managing solicited and spontaneously reported adverse events and other unintended effects of trial interventions or trial conduct	-	

For peer review only

SPIRIT | CONSORT OUTCOMES Reporting Guidelines for Trial Outcomes

Section	Item No.	SPIRIT 2013 Item	SPIRIT-Outcomes 2022 item	Location Reported[b]
Auditing	23	Frequency and procedures for auditing trial conduct, if any, and whether the process will be independent from investigators and the sponsor	-	
**Ethics and dissemination**				
Research ethics approval	24	Plans for seeking research ethics committee/institutional review board (REC/IRB) approval	-	
Protocol amendments	25	Plans for communicating important protocol modifications (eg, changes to eligibility criteria, outcomes, analyses) to relevant parties (eg, investigators, REC/IRBs, trial participants, trial registries, journals, regulators)	-	
Consent or assent	26a	Who will obtain informed consent or assent from potential trial participants or authorised surrogates, and how (see Item 32)	-	
	26b	Additional consent provisions for collection and use of participant data and biological specimens in ancillary studies, if applicable	-	
Confidentiality	27	How personal information about potential and enrolled participants will be collected, shared, and maintained in order to protect confidentiality before, during, and after the trial	-	
Declaration of interests	28	Financial and other competing interests for principal investigators for the overall trial and each study site	-	
Access to data	29	Statement of who will have access to the final trial dataset, and disclosure of contractual agreements that limit such access for investigators	-	
Ancillary and post-trial care	30	Provisions, if any, for ancillary and post-trial care, and for compensation to those who suffer harm from trial participation	-	
Dissemination policy	31a	Plans for investigators and sponsor to communicate trial results to participants, healthcare professionals, the public, and other relevant groups (eg, via publication, reporting in results databases, or other data sharing arrangements), including any publication restrictions	-	
	31b	Authorship eligibility guidelines and any intended use of professional writers	-	

For peer review only - http://bmjopen.bmj.com/site/about/guidelines.xhtml

Section	Item No.	SPIRIT 2013 Item	SPIRIT-Outcomes 2022 item	Location Reported[b]
	31c	Plans, if any, for granting public access to the full protocol, participant-level dataset, and statistical code	-	
**Appendices**				
Informed consent materials	32	Model consent form and other related documentation given to participants and authorised surrogates	-	
Biological specimens	33	Plans for collection, laboratory evaluation, and storage of biological specimens for genetic or molecular analysis in the current trial and for future use in ancillary studies, if applicable	-	

[a]It is strongly recommended that this checklist be read in conjunction with the SPIRIT (Standard Protocol Items: Recommendations for Interventional Trials) Statement paper for important clarification on the items. Amendments to the protocol should be tracked and dated. The SPIRIT checklist is copyrighted by the SPIRIT Group under the Creative Commons "Attribution-NonCommercial-NoDerivs 3.0 Unported" license and is reproduced with permission.
[b]Indicates page numbers and/or manuscript location: to be completed by authors.

For peer review only

For peer review only - http://bmjopen.bmj.com/site/about/guidelines.xhtml

The Newcastle upon Tyne Hospitals
NHS Foundation Trust

Principal Investigator: Dr Silvia Del Din

Participant Identification Number:

Participant Consent Form

Confidence in Concept (CiC) - Translating digital healthcare to enhance clinical management: evaluating the effect of medication on mobility in people with Parkinson's disease (PD).

We are inviting you to take part in a medical research study.
Please make sure you have read the accompanying Participant Information Sheet which explains why we are doing this research and what we are asking you to do.
Please ask a member of the research team if there is anything that is not clear or if you would like more information.
It is entirely up to you if would like to take part or not.

For peer review only

For peer review only - http://bmjopen.bmj.com/site/about/guidelines.xhtml

CiC – Effect of medication on mobility in people with PD
Consent Form V2.1 – 14 May 2021
IRAS ID: 295771

NHS
The Newcastle upon Tyne Hospitals
NHS Foundation Trust

	Please initial box
I confirm that I have read and understand the Participant Information Sheet (version and date ……………………) for the above study. I have had the opportunity to consider the information, ask questions and have had these answered satisfactorily.	
I understand that my participation is voluntary and that I am free to withdraw at any time without giving any reason, without my medical care or legal rights being affected.	
I understand that relevant sections of my medical notes and data collected during the study may be looked at by individuals from local clinical care team, from Newcastle University, from regulatory authorities or from the NHS Trust, where it is relevant to my taking part in this research. I give permission for these individuals to have access to my records.	
I understand that the anonymised information collected about me will be stored and used to support other research in the future, and may be shared anonymously with other researchers.	
In the event that a new potential medical problem is found as a result of my participation, I agree that the research team may inform my GP or relevant medical professional about this following a discussion with myself.	
I understand that I will be provided with a wearable technology system comprising of an activity monitor, a smartwatch and a smartphone which will be worn and used for seven days following the visit.	
I agree to participate in this study.	

Name of Participant	Date	Signature
Name of Person Taking Consent	Date	Signature

For peer review only

Healthcare at its best
with people at our heart

The Newcastle upon Tyne Hospitals
NHS Foundation Trust

Please ensure that a copy is offered to the participant or relative/carer and record whether this offer was accepted/ declined.

Copy given [ ]	Copy declined [ ]

Original to be filed in the Trial Master File. Copy to be included in the participant medical records.

For peer review only

For peer review only - http://bmjopen.bmj.com/site/about/guidelines.xhtml

NHS
The Newcastle upon Tyne Hospitals
NHS Foundation Trust

Principal Investigator: Dr Silvia Del Din

Participant Identification Number:

Participant Consent Form Consent Form for Mobilise-D – Clinical Validation Sub-Study

Confidence in Concept (CiC) - Translating digital healthcare to enhance clinical management: evaluating the effect of medication on mobility in people with Parkinson's disease (PD).

As a participant in the Mobilise-D - Clinical Validation Study, we are asking you to volunteer to take part in additional data collection for a sub-study.

Please make sure you have read the accompanying Participant Information Sheet which explains why we are doing this research and what we are asking you to do.

Please ask a member of the research team if there is anything that is not clear or if you would like more information.

It is entirely up to you if would like to take part or not in this additional data collection.

For peer review only - http://bmjopen.bmj.com/site/about/guidelines.xhtml

CiC – Effect of medication on mobility in people with PD
Consent Form V2.1 - 14 May 2021
IRAS ID: 295771

NHS
The Newcastle upon Tyne Hospitals
NHS Foundation Trust

	Please initial box
I confirm that I have read and understand the Participant Information Sheet (version and date ……………………) for the above study. I have had the opportunity to consider the information, ask questions and have had these answered satisfactorily.	
I understand that my participation is voluntary and that I am free to withdraw at any time without giving any reason, without my medical care or legal rights being affected.	
I understand that relevant sections of my medical notes and data collected during the study may be looked at by individuals from local clinical care team, from Newcastle University, from regulatory authorities or from the NHS Trust, where it is relevant to my taking part in this research. I give permission for these individuals to have access to my records.	
I understand that the anonymised information collected about me will be stored and used to support other research in the future, and may be shared anonymously with other researchers.	
In the event that a new potential medical problem is found as a result of my participation, I agree that the research team may inform my GP or relevant medical professional about this following a discussion with myself.	
I understand that as part of the Mobilise-D Clinical Validation Study I will be provided with an activity monitor which will be worn for seven days following the baseline visit and that, for this additional data collection, I will be also provided with a smartwatch and a smartphone which will be used during the same seven day period.	
I agree to participate in this sub-study and additional data collection.	

Name of Participant Date Signature

Name of Person Taking Consent Date Signature

For peer review only - http://bmjopen.bmj.com/site/about/guidelines.xhtml

Please ensure that a copy is offered to the participant or relative/carer and record whether this offer was accepted/ declined.

Copy given [ ]	Copy declined [ ]

Original to be filed in the Trial Master File. Copy to be included in the participant medical records.

For peer review only

For peer review only - http://bmjopen.bmj.com/site/about/guidelines.xhtml

CiC – Effect of medication on mobility in people with PD

Version 1.1; 14 May 2021; IRAS ID: 295771

Subject Initial |__||__|
F S

Subject ID |__|__|__|__|__|

**Day/ Time**	**6:00 7:00**	**7:00 8:00**	**8:00 9:00**	**9:00 10:00**	**10:00 11:00**	**11:00 12:00**	**12:00 13:00**	**13:00 14:00**	**14:00 15:00**	**15:00 16:00**	**16:00 17:00**	**17:00 18:00**	**18:00 19:00**	**19:00 20:00**	**20:00 21:00**	**21:00 22:00**	Comments
Monday																	
Tuesday																	
Wednesday																	
Thursday																	
Friday																	
Saturday																	
Sunday																	

*Split cells indicate the "half an hour" – e.g. 6.30

Example: Off status ("O") between 6.30 and 7.00 and dyskinesia ("D") between 7.00 and 7.30 on Monday

**Day/ Time**	**6:00 7:00**		**7:00 8:00**		**8:00 9:00**	
Monday		O	D			

For peer review only

CiC – Effect of medication on mobility in people with PD
Version 1.1; 14 May 2021; IRAS ID: 295771

**Subject Initial**	**Subject ID**
|___||___| (F, S)	|___|___|___|___|___|

Usability questionnaire

For peer review only

Subject Initials |__||__|
F S

Subject ID |__|__|__|__|__|

English versions of questionnaires

Rabinovich

Please select a response for each of these questions that best reflects your experiences and opinions of the Wearable Technology System (Wearable device, smartwatch and smartphone).

Section A

How much trouble did you have getting started with the Wearable Technology System?

(NOTE: 'getting started with' refers to the first few hours of wear/use in the week. It is how the participant felt while using/wearing it initially at the start of the week)

O No trouble to start up

O Sometimes trouble to start up

O Regularly caused trouble to start up

O Always trouble to start up

O I had to call the centre to get help in starting-up

The Wearable Technology System was easy to put on/take off

O Yes this was very easy

O This worked just fine

O I found it somewhat difficult

O I found it difficult

O I was unable to manage this on my own

I experienced technical problems with the Wearable Technology System

O All the time

O Frequently

O Sometimes

O Seldom

O Never

For peer review only

CiC – Effect of medication on mobility in people with PD

Subject Initials |__||__|
F S

Subject ID |__|__|__|__|__|

The Wearable Technology System interfered with my normal activities

O All the time

O Frequently

O Sometimes

O Occasionally

O Never

I felt comfortable wearing the Wearable Technology System

O All the time

O Frequently

O Sometimes

O Occasionally

O Never

I felt embarrassed wearing the Wearable Technology System

O All the time

O Frequently

O Sometimes

O Occasionally

O Never

The instructions on how to use the Wearable Technology System were clear

O Strongly disagree

O Disagree

O Neutral

O Agree

O Strongly agree

For peer review only

Subject Initials |__||__|
F S

Subject ID |__|__|__|__|__|

Using the Wearable Technology System on a daily basis was easy

O Strongly disagree

O Disagree

O Neutral

O Agree

O Strongly agree

The Wearable Technology System was bulky/heavy.

O Yes , very much so

O Yes much

O Not particularly

O Not at all

O No opinion

The Wearable Technology System (Wearable device) bothered me in the bed.

O Yes , very much so

O Yes much

O Not particularly

O Not at all

O No opinion, I did not wear the monitor at night

I felt my privacy was invaded by the Wearable Technology System

O Strongly disagree

O Disagree

O Neutral

O Agree

O Strongly agree

For peer review only

CiC – Effect of medication on mobility in people with PD

Subject Initials |__||__|
F S

Subject ID |__|__|__|__|__|__|

<u>If my doctor would like to use the Wearable Technology System to assess my activity and medication adherence I would be willing to wear it and use it for</u>

O Less than 1 day

O 2-4 days

O 1week

O Longer than 1 week.

O I would not mind wearing the monitor continuously (longer than 1 month)

Section B

We want to ask you to provide a final score for the Wearable Technology System.

All things considered can you give the device a score from 0% to 100%, where 0 means the worst possible system and 100% means the ideal system in your opinion.

<u>My score for Wearable Technology System</u>: .... /100

In the section below we want to give you the opportunity to give other comments on the Wearable Technology System and its component (wearable device (lower back), smartwatch and smartphone).

Wearable device:

Smartwatch:

Smartphone:

WT as a whole:

I experienced the following problems with the Wearable Technology System:

Wearable device:

Smartwatch:

Smartphone:

WT as a whole:

I liked these features of the Wearable Technology System in particular:

Wearable device:

Smartwatch:

Smartphone:

WT as a whole:

For peer review only

BMJ Open

Translating digital healthcare to enhance clinical management: a protocol for an observational study utilising a digital health technology system to monitor medication adherence and its effect on mobility in people with Parkinson’s

Journal:	*BMJ Open*
Manuscript ID	bmjopen-2023-073388.R1
Article Type:	Protocol
Date Submitted by the Author:	10-Jul-2023
Complete List of Authors:	Packer, Emma; Newcastle University Translational and Clinical Research Institute, Faculty of Medical Sciences<br>Debelle, Héloïse; Newcastle University Translational and Clinical Research Institute, Faculty of Medical Sciences<br>Bailey, Harry; Newcastle University Translational and Clinical Research Institute, Faculty of Medical Sciences<br>Ciravegna, Fabio; Università degli Studi di Torino Dipartimento di informatica<br>Ireson, Neil; The University of Sheffield, Department of Computer Science and Insigneo Institute<br>Evers, Jordi; McRoberts BV<br>Niessen, Martijn; McRoberts BV<br>Shi, Jian Qing; Southern University of Science and Technology; National Center for Applied Mathematics<br>Yarnall, Alison J; Newcastle Upon Tyne Hospitals NHS Foundation Trust; Newcastle University Translational and Clinical Research Institute, Faculty of Medical Sciences<br>Rochester, Lynn; Newcastle Upon Tyne Hospitals NHS Foundation Trust; Newcastle University Translational and Clinical Research Institute, Faculty of Medical Sciences<br>Alcock, Lisa; Newcastle University Translational and Clinical Research Institute, Faculty of Medical Sciences; NIHR Newcastle Biomedical Research Centre, based at The Newcastle upon Tyne Hospitals NHS Foundation Trust, Newcastle University<br>Del Din, Silvia; Newcastle University Translational and Clinical Research Institute, Faculty of Medical Sciences; NIHR Newcastle Biomedical Research Centre, based at The Newcastle upon Tyne Hospitals NHS Foundation Trust, Newcastle University
**Primary Subject Heading**:	Research methods
Secondary Subject Heading:	Diagnostics, Medical management, Geriatric medicine
Keywords:	Parkinson-s disease < NEUROLOGY, Quality of Life, Protocols & guidelines < HEALTH SERVICES ADMINISTRATION & MANAGEMENT, Aging

SCHOLARONE™
Manuscripts

Translating digital healthcare to enhance clinical management: a protocol for an observational study utilising a digital health technology system to monitor medication adherence and its effect on mobility in people with Parkinson's

Emma Packer (0000-0002-0491-3726), Héloïse Debelle (0000-0001-9455-7448), Harry G.B Bailey (0000-0002-4442-293X), Fabio Ciravegna (0000-0001-5817-4810), Neil Ireson (0009-0009-2278-5180), Jordi Evers (0000-0002-7970-8575), Martijn Niessen (0000-0003-3515-4731), Jian Qing Shi (0000-0002-2924-1137), Alison J Yarnall (0000-0002-3126-9163), Lynn Rochester (0000-0001-5774-9272), Lisa Alcock (0000-0002-8364-9803), Silvia Del Din* (0000-0003-1154-4751)

Translational and Clinical Research Institute, Faculty of Medical Sciences, Newcastle University, Newcastle upon Tyne, UK, Emma Packer, PhD Student

Translational and Clinical Research Institute, Faculty of Medical Sciences, Newcastle University, Newcastle upon Tyne, UK, Héloïse Debelle, Research Assistant

Translational and Clinical Research Institute, Faculty of Medical Sciences, Newcastle University, Newcastle upon Tyne, UK, Harry G.B Bailey, Research Assistant

Dipartimento di Informatica, Univsersità di Torino, Italy, Fabio Ciravegna, Professor of Pervasive Technologies

Department of Computer Science and INSIGNEO Institute for in silico Medicine, The University of Sheffield, Sheffield, UK, Neil Ireson, Research Associate

McRoberts BV, The Hague, The Netherlands, Jordi Evers, Chief Marketing Officer

McRoberts BV, The Hague, The Netherlands, Martijn Niessen, Chief Executive Officer

Department of Statistics and Data Science, Southern University of Science and Technology, Shenzhen, China | National Center for Applied Mathematics Shenzhen, China, Jian Qing Shi, Professor of Statistics

Translational and Clinical Research Institute, Faculty of Medical Sciences, Newcastle University, Newcastle upon Tyne, UK | The Newcastle Upon Tyne Hospitals NHS Foundation Trust, Newcastle upon Tyne, UK, Alison J Yarnall, NIHR BRC Clinical Senior Lecturer and Honorary Consultant in Older People's Medicine

Translational and Clinical Research Institute, Faculty of Medical Sciences, Newcastle University, Newcastle upon Tyne, UK | The Newcastle Upon Tyne Hospitals NHS Foundation Trust, Newcastle upon Tyne, UK, Lynn Rochester, Professor of Human Movement Science

Translational and Clinical Research Institute, Faculty of Medical Sciences, Newcastle University, Newcastle upon Tyne, UK | National Institute for Health and Care Research (NIHR) Newcastle Biomedical Research Centre (BRC), based at Newcastle upon Tyne Hospitals NHS Foundation Trust, Newcastle University and the Cumbria, Northumberland and Tyne and Wear (CNTW) NHS Foundation Trust, Lisa Alcock, Senior Research Associate

For peer review only

For peer review only - http://bmjopen.bmj.com/site/about/guidelines.xhtml

Translational and Clinical Research Institute, Faculty of Medical Sciences, Newcastle University, Newcastle upon Tyne, UK | National Institute for Health and Care Research (NIHR) Newcastle Biomedical Research Centre (BRC), based at Newcastle upon Tyne Hospitals NHS Foundation Trust, Newcastle University and the Cumbria, Northumberland and Tyne and Wear (CNTW) NHS Foundation Trust, Silvia Del Din, Newcastle University Academic Track Fellow

* Corresponding author

Corresponding author

Dr Silvia Del Din

Translational and Clinical Research Institute

Faculty of Medical Sciences

The Catalyst

3 Science Square, Room 3.27

NE4 5TG

Email: Silvia.Del-Din@newcastle.ac.uk

Phone: +44 (0) 191 208 1131

For peer review only

Word count: 3988 (excluding title page, abstract, article summary, footnotes, contributions, acknowledgements, references, figures, and tables)

Key words: Wearable Technology, Parkinson's disease, Protocol, Human, Walking, Medication Adherence.

ABSTRACT

Introduction: In people with Parkinson's (PwP) impaired mobility is associated with an increased falls risk. To improve mobility, dopaminergic medication is typically prescribed, but complex medication regimens result in sub-optimal adherence. Exploring medication adherence and its impact on mobility in PwP will provide essential insights to optimise medication regimens and improve mobility. However, this is typically assessed in controlled environments, during one-off clinical assessments. Digital health technology (DHT) presents a means to overcome this, by continuously and remotely monitoring mobility and medication adherence. This study aims to utilise a novel digital health technology system (DHTS) (comprising of a smartphone, smartwatch, and Inertial Measurement Unit (IMU)) to assess self-reported medication adherence, and its impact on digital mobility outcomes (DMOs) in PwP.

Methods and analysis: This single-centre, UK-based study, will recruit 55 participants with Parkinson's. Participants will complete a range of clinical, and physical assessments. Participants will interact with a DHTS over seven-days, to assess self-reported medication adherence, and monitor mobility and contextual factors in the real-world. Participants will complete a motor complications diary (ON-OFF-Dyskinesia) throughout the monitoring period and, at the end, a questionnaire and series of open-text questions to evaluate DHTS usability. Feasibility of the DHTS and the motor complications diary will be assessed. Validated algorithms will quantify DMOs from IMU walking activity. Time series modelling and deep learning techniques will model and predict DMO response to medication and effects of contextual factors. This study will provide essential insights into medication adherence and its effect on real-world mobility in PwP, providing insights to optimise medication regimens.

Ethics and dissemination: Ethical approval was granted by London - 142 Westminster Research Ethics Committee (REC: 21/PR/0469), protocol v2.4. Results will be published in peer-reviewed journals. All participants will provide written, informed consent.

Study registration number: ISRCTN (13156149)

Article Summary

Strengths and limitations of this study

- This protocol has been developed by a multidisciplinary team of experienced clinicians, researchers, and data analysts.
- This study will monitor mobility and assess self-reported medication adherence in the real-world, enhancing the ecological validity of results.
- If efficacious, this protocol may be implemented in clinical settings to optimise medication regimens and enhance the quality of life for people with Parkinson's disease.
- Participants will only be required to attend a single clinic-based assessment, with the remainder of the study conducted in their home or community.
- This study will include participants with mild to moderate disease severity, therefore, our conclusions may not reflect the experience of those with more advanced Parkinson's disease.

For peer review only - http://bmjopen.bmj.com/site/about/guidelines.xhtml

INTRODUCTION

Mobility, defined as the ability to freely move about one's environment, is a key contributor to overall health and maintaining independence (1, 2). People with Parkinson's (PwP) experience a loss of mobility due to the cardinal motor symptoms of bradykinesia, rigidity, tremor, and postural instability (3). This loss of mobility is associated with an increased risk of falls and reduced independence; therefore, mitigating mobility loss in PwP is of primary importance (4).

In PwP, dopaminergic medications (e.g. Levodopa) are commonly prescribed to alleviate motor symptoms and improve mobility (3). Medication is often prescribed in multiple doses throughout the day, with adherence to prescribed dosage and timing essential to alleviate motor symptoms and avoid fluctuations, especially as the disease progresses. Complex medication regimens result in suboptimal adherence, poor medication responses, and reduced quality of life (5-7). Modification to regimens often follows patient-reported outcomes and short, infrequent clinical appointments, with clinicians observing patients during specific stages in their regimen (e.g., ON/OFF periods). Consequently, clinicians often lack adequate insight into patient's medication habits and daily fluctuations in motor symptoms to appropriately adapt regimens. Understanding medication adherence in PwP and its effect on their everyday mobility and motor symptoms would ensure more effective and personalised patient management, allowing clinicians to optimise dose and frequency of dopaminergic drugs.

At present, research exploring medication and mobility in PwP is typically conducted in controlled lab-based or clinical environments, in specific ON/OFF medication states (8-16). Although some studies have been performed in the real-world (i.e., daily living, outside clinical or laboratory settings) (17, 18), a more granular picture is required. Digital health technology (DHT) presents the tools to explore this, by allowing for the continuous assessment of medication adherence and mobility (digital mobility outcomes, DMOs) in the real-world (5, 19-22). A variety of DMOs can be utilised to assess mobility; and of primary interest is walking speed (23). Walking speed reflects overall health in PwP and declines overtime, with slow walking speed associated with adverse clinical events such as an increased risk of falls (4); therefore, assessing this DMO can provide insights into medication related changes in overall mobility and health. The COVID-19 pandemic has also highlighted the fragility of clinic-based care for PwP and reinforced the necessity to utilise DHT to improve current practice (24).

There are a variety of DHTs which can monitor individuals during their day-to-day lives. For example, the Personal KineticGraph (PKG, Global kinetics Corp, Australia), a wrist-worn device, can send medication reminders and continuously monitor motor symptoms such as bradykinesia, tremor, and dyskinesia (25, 26). However, technology such as this cannot monitor the specific medication ingested or quantify gait characteristics. Future research should therefore focus on developing a DHT system (DHTS) which can comprehensively and remotely assess medication adherence and monitor mobility. Data collected using this system would allow for the creation of data driven models to investigate variations in mobility following medication intake, thus providing greater insights for optimised medication regimens in PwP.

This study aims to collect real-world data using a novel DHTS (smartphone, smartwatch, and inertial measurement unit) to assess self-reported medication adherence, quantify DMOs, and model and predict their response to medication. Within this study, specific objectives include: (i) clinically characterise our participants with Parkinson's (e.g., Hoehn and Yahr stage, disease duration etc.); (ii) collect real-world data with a connected DHTS in PwP; (iii) assess real-world, self-reported medication adherence in PwP by recording medication intakes (smartwatch reminders, time of logged intakes and acknowledged

For peer review only - http://bmjopen.bmj.com/site/about/guidelines.xhtml

medication intake events); (iv) quantify real-world DMOs (e.g., walking speed) and (v) model and predict response of DMOs to Parkinson's medication.

METHODS AND ANALYSIS

The SPIRIT reporting guidelines have been followed within this manuscript (27) (supplementary material 1). This protocol represents protocol version 2.4, April 2022.

Study design and setting

This cross-sectional, observational study will recruit 55 participants with Parkinson's disease (IRAS Number: 295771). It will be conducted at the Clinical Ageing Research Unit, Campus for Ageing and Vitality, Newcastle University, Newcastle upon Tyne, NE4 5PL, with a remote home and community-based component. The study is co-funded by the Medical Research Council (MRC) Newcastle University, the United Kingdom Research Innovation, Engineering and Physical Sciences Research Council (UKRI ESPRC) and the National Institute of Health Research (NIHR). This study is a sub-study of the 'Mobilise-D – Clinical Validation Study' (IRAS Number: 289543 and REC reference: 20/PR/0792), a longitudinal study, which aims to validate a novel digital method for remote monitoring of mobility (28).

Study procedure and flow

Recruitment and screening

In this single-centre study, participants will be identified and recruited between June 2021 – June 2024 through the "Mobilise-D - Clinical Validation Study" Newcastle upon Tyne site, and independently through local movement disorder clinics and research registries. As recruitment commenced in the height of the COVID-19 pandemic - i.e., when individuals were less mobile, less likely to partake in research, and there was insecurity surrounding future lockdowns - an extended recruitment period was afforded to this study. This was to ensure to our sample size was achieved and results were representative. Interested and potentially eligible participants will attend a screening appointment during which informed consent will be obtained, and eligibility confirmed. Informed consent will be taken by the Chief Investigator or a delegated member of the research team. At the beginning of the screening appointment and prior to further discussion, the individual obtaining consent will confirm that the potential participant has read the Participant Information Sheet (PIS), and will answer any questions. Upon agreement to participate in the study, the participant will sign and date the informed consent form and the researcher will witness this, and sign and date the same form (supplementary material 2 and 3). The process will then be recorded in the participants' medical records, with the original filed on site and a copy given to the participant. Those recruited through the Mobilise-D study will attend a screening appointment within a Mobilise-D visit and those recruited independently will attend a separate screening appointment. A full outline of the recruitment procedure is detailed in Figure 1. All participants will be reimbursed for travel expenses.

Due to the paucity of research exploring the influence of medication on real-world mobility in PwP using a DHTS, there was insufficient data to inform a reliable power calculation before the study commenced. Therefore, a convenience sample size of 55 was defined according to COnsensus-based Standards for selection of health Measurement Instruments (COSMIN) guidelines (29), with the plan to assess this once 50% of data was collected. The sample size was evaluated using data collected in 30 participants, with GPower v3.1, assuming an effect size of 0.44 for a change in mobility (gait speed, shown to be improved by levodopa in lab studies (16) related to off vs on state (repeated paired t-test, alpha of 0.05 and beta (power) of 0.95). This highlighted that our sample size of 55 participants was appropriate.

Please insert Figure 1 here.

Figure 1 caption: 'Figure 1. Flow chart of recruitment and screening procedure.'

For peer review only - http://bmjopen.bmj.com/site/about/guidelines.xhtml

Participants and eligibility criteria

Participants with mild to moderate disease severity will be recruited (Hoehn and Yahr stage Ⅰ-Ⅲ). The eligibility criteria (inclusion and exclusion) are in agreement with the Mobilise-D study and are detailed in Table 1. Potential participants will not be recruited if they have received and/or are due to receive a medication review within four weeks of the study assessment. Individuals with more advanced Parkinson's (Hoehn and Yahr stage IV and V) often possess a decreased ability to efficiently perform activities of daily living and experience cognitive impairment. These factors may induce difficulties to monitor mobility and alter the participant's ability to interact with the DHTS, respectively. Consequently, only individuals at Hoehn and Yahr stages I-III will be recruited in this study.

Table 1. Inclusion and Exclusion Criteria.

Inclusion criteria	Exclusion criteria
• Adults aged 18 or over	• Occurrence of any of the following within 3 months prior to informed consent: myocardial infarction, hospitalisation for unstable angina, stroke, coronary artery bypass graft, percutaneous coronary intervention, implantation of a cardiac resynchronization therapy device, active treatment for cancer or other malignant disease, uncontrolled congestive heart disease (NYHA class >3), acute psychosis, major psychiatric disorders, or continued substance abuse
• Ability to consent and comply with study specific procedures	• History consistent with Dementia with Lewy Bodies, atypical parkinsonian syndromes (including multiple system atrophy or progressive supranuclear palsy, diagnosed according to accepted criteria)
• Able to read and write in English	• Repeated strokes or stepwise progression of symptoms, leading to a diagnosis of 'vascular parkinsonism'
• Patients with the clinical diagnosis of Parkinson's according to the Movement Disorder Society criteria (30)	• Drug-induced Parkinsonism
• Hoehn and Yahr stage I – III (31)	
• On stable Parkinson's medication doses (taking the same medication for four weeks or more)	
• Able to walk 4 metres independently with or without walking aids	
• Willingness to wear and interact with a smartwatch and IMU, and carry a smartphone	

For peer review only - http://bmjopen.bmj.com/site/about/guidelines.xhtml

Study assessments

At the beginning of the study, all participants will complete a clinic-based assessment including a range of general, clinical, Parkinson's-specific, and physical assessments (Table 2). These assessments will be performed during a Mobilise-D visit or within 14 days of the screening appointment for those recruited independently. Following assessment completion, participants will be equipped with a novel DHTS to wear and interact with over seven consecutive days and will complete a motor complications diary (ON-OFF-Dyskinesia) each day. Participants will be free to withdraw from the study at any time, without providing reasoning and they will be given a contact point to obtain further study information.

Researchers who collect data pertaining to outcome measures will receive appropriate training before data collection (e.g., Good Clinical Practice training, informed consent in clinical research training, MDS-UPDRS and Montreal Cognitive Assessment training). To promote participant recruitment and retention, participants will be given detailed oral and written instructions via a PIS. The PIS will include details for day-to-day use of the devices (i.e., how to charge the devices, how to respond to medication notifications etc.) and contact details for the research team should any issues or questions arise.

Table 2. Outcome measures.

**General measures**
• Descriptive measures (year of birth, sex, living arrangement, employment status, years in education, native language, marital status)
• Anthropometric measures (height, mass, shoe size, and leg length)
• Health status (current medication and use of mobility aids (e.g., walking frame))
**Clinical measures**
• Disease Duration
• Late-Life Functional Disability Instrument (LLFDI) (32)
• Levodopa equivalent daily dose (LEDD) (33, 34)
• Frailty Phenotype (35)
**Parkinson's disease specific measures**
• Movement Disorder Society – Unified Parkinson's Disease Rating Scale (MDS-UPDRS) (30)
• Hoehn and Yahr Stages (31)
• New Freezing of Gait Questionnaire (NFOG-Q) (36)
• Montreal Cognitive Assessment (MoCA) (37)
**Physical Measures**
• Short Physical Performance Battery (SPPB) (38)
• Seven-day remote monitoring period (interact with DHTS)
**Diary, questionnaire, and qualitative questions**
• Motor complications diary (ON-OFF-dyskinesia)
• Usability questionnaire (39)
• Open-text questions

For peer review only

For peer review only - http://bmjopen.bmj.com/site/about/guidelines.xhtml

Seven-day remote monitoring period

Primary outcome measures include self-reported medication adherence (i.e. frequency, intake time, and dosage of medication) captured by the smartwatch and walking speed, quantified via the IMU. Secondary outcome measures include macro outcomes (related to volume (e.g. step count), pattern (e.g. walking bout length), and variability (e.g. walking bout length variability) of walking activity and micro outcomes (related to pace (e.g., gait speed), rhythm (e.g., step time), variability (e.g., step time variability), asymmetry (e.g., step time asymmetry), and postural control (e.g. step length asymmetry)) (40-43). DMOs obtained during the seven-day monitoring period. In addition, contextual factors captured on the smartphone, including geolocation (probability of being indoors/outdoors), number of steps outside the home, stay points, common paths, and weather conditions (for more detail, see Smartphone section below).

Measurement tools and data collection

To collect data on self-reported medication adherence, DMOs, and contextual insights, participants will interact with the novel DHTS (smartphone, smartwatch, and IMU) over seven consecutive days. This monitoring period will commence following a Mobilise-D appointment or following the initial assessment for those recruited independently.

Smartphone to collect contextual data

As this study will be carried out remotely, in the participant's home and their community, contextual factors such as weather conditions and being indoors or outdoors, may influence DMOs. Therefore, capturing these contextual factors will help better describe, understand and control for these aspects when looking at the effect of medication on DMOs. To quantify the effect of these confounding contextual factors on DMOs, participants will be advised to carry a smartphone (Samsung Galaxy S9, S10 or S21) whenever outside their house, making sure it is switched on at all times and fully charged. Participants will be provided with a charger and will be advised to charge the smartphone every night throughout the monitoring period.

The custom-made Aeqora application extension will be pre-installed onto each smartphone (Department of Computer Science, The University of Sheffield, UK). This application will utilise the phone's sensors to capture contextual information, such as, geolocation, stay points, common paths, number of steps, and weather conditions. The application has been developed as an Android mobile application. It is made up of three components: (i) the core tracker, (ii) the interface, and (iii) the server infrastructure, which collects data across users (44). The core tracker has been adapted from a library developed by the University of Sheffield (45). This tracker identifies the type of activity (e.g., walking) from the phone's internal sensors (accelerometer and gyroscope). It continually operates in the background to sense mobility features using these sensors and location services, such as Bluetooth (44). To identify the weather conditions at the time of measurement, the closest weather station to the participant's location will be utilised. Raw sensor data will be collected and stored in real-time onto a local database which is synchronised with a remote database when the phone is recharging. No participant identifiable information will be sent to the server. The data is synched to the remote database via a Secure Socket Layer (SSL) and Transports Layer Security (TLS) 3.0 protocol. To ensure privacy and security when communicating sensitive data an SSL/TSL certificate is exchanged between client and server, which establishes the identity and trust between them.

Smartwatch to assess real-world, self-reported medication adherence

To assess real-world, self-reported medication adherence, participants will wear and interact with a smartwatch (TicWatch Pro 3, Mobvoi) throughout the monitoring period. During the clinic visit, a

For peer review only - http://bmjopen.bmj.com/site/about/guidelines.xhtml

researcher will manually input the participant's medication reminders onto the Aeqora application. This application will then send notifications from the smartphone to the smartwatch to remind participants of their Parkinson's-specific medication intake times and allow them to log the times at which their medication was taken, clicking either "YES" or "NO" on the watch face when prompted (Figure 2). The watch will also allow participants to log any additional Parkinson's-specific medication they have taken ('as required'). Participants will be provided with a smartwatch charger and will be required to remove and charge the smartwatch overnight throughout the monitoring period. Common, validated methods for monitoring medication adherence rely on questionnaires and diary completion. These place extra burden onto participants and are often subjective. For this reason, this study utilises the objective method of responding to notifications on a smartwatch, as this provides objective, real-time insights into medication adherence, and minimises participant-burden.

IMU to quantify digital mobility outcomes

To quantify primary and secondary DMOs, participants will continuously wear an IMU (Axivity AX6, Axivity Ltd, Newcastle upon Tyne, UK, including triaxial accelerometer and gyroscope, dimensions 23 x 32.5 x 8.9mm, mass 11g, frequency 100Hz, gyroscope range ± 2000 degrees per second and accelerometer range ± 8g) throughout the monitoring period. The IMU will be secured to the participant's lower back, at the fifth lumbar vertebrae (44). It will be affixed using a hydrogel/hydrocolloid adhesive and a hypoallergenic plaster patch. Participants will be advised to continue with their usual activities and not change their routine. Throughout the monitoring period, all IMU data will be stored locally on the IMU hardware. After the monitoring period, participants will return the IMUs via pre-paid envelopes and data will be downloaded and uploaded onto the eScience (e-SC) (a cloud based storage platform) CiC Data Management platform (46, 47) (see data management section). The IMU will be worn at all times, including during the night and although the IMU is waterproof participants will be advised to remove it during prolonged water exposure (e.g., swimming, taking a bath).

Please insert Figure 2 here.

Figure 2 caption: 'Figure 2. Digital Health Technology System (DHTS). (A) Inertial Measurement Unit (IMU), which monitors mobility. (B) Smartwatch which assesses self-reported medication adherence, by notifying participants when to take their medication and allowing them to log the time at which they took their medication. (C) Smartphone which collects contextual data.'

Diary to monitor motor complications

During the seven-day monitoring period, participants will fill in a motor complications diary (paper-based) for each day, indicating their "OFF-status" (when they feel their medication is not working) and whether they experienced dyskinesia (involuntary movements). The diary will record data over 16 hours each day from 6:00am to 22:00pm, from days one to seven (supplementary material 4).

Assessment of participants' experience with the DHTS

Questionnaire to evaluate usability of the DHTS

To evaluate the participants' experience with the DHTS, participants will complete an adapted version of Rabinovich et al's 12-item usability questionnaire at the end of the monitoring period (supplementary material 5) (39). This questionnaire investigates usability on a 5-point ordinal scale, from 5 (most favourable) to 1 (least favourable), and 'no opinion' scored as 3. The questions identify participant's attitude towards the DHTS, its ease of use and comfort, such as, "I experienced technical problems" and "The monitors interfered with my normal activity". Participants will also rate the DHTS on a scale from 0 (worst score) to 100 (best score).

For peer review only - http://bmjopen.bmj.com/site/about/guidelines.xhtml

Qualitative feedback on the DHTS and its individual devices will be collected via open-text questions added to the end of the questionnaire (supplementary material 5). These questions require participants to "give any other comments on the DHTS and its components", to describe the "problems" they experienced when using the DHTS and its components, as well as any features they liked about the system and its individual devices. All devices will be returned at the end of the assessment period, in addition to the questionnaire and motor complications diary, using pre-paid tracked envelopes.

Feasibility of DHTS and motor complications diary

To assess whether the DHTS and motor complications diary are feasible for everyday use in PwP, this study will assess their feasibility (see data analysis section for details).

Patient and Public Involvement

As this study is a sub-study of the "Mobilise-D – Clinical Validation Study" insights from patient and public advisory groups which informed the Mobilise-D study were used to inform the development of this study. The Mobilise-D Study Participant Information Sheet (PIS) was reviewed and modified by relevant patient groups and was used as a guide for this study's PIS. Participants in this study will provide feedback on usability of the DHTS, which will inform future studies. Study insights will be fed back to patient stakeholder groups within the Mobilise-D consortium.

Data and Statistical Analysis

Data analysis

To quantify DMOs, mobility data from the IMUs will be analysed using validated Matlab® algorithms (R2019a, Mathworks, California, United States) (47, 48). To investigate the effect of medication on DMOs, medication intake timestamps (captured via the smartwatch) and mobility data from the IMU will be utilised. The effect of contextual factors (e.g., weather conditions and probability of being indoors/outdoors) on DMOs will be considered when interpreting the medication and mobility data.

Responses to open-text questions will be analysed using a hybrid approach, with both deductive and inductive methods used to analyse responses. Responses will be grouped into three themes: feasibility, usability, and recommendations for improvement. Once grouped, key sub-themes within each group (i.e., ease of use for the usability theme) will be identified following the same approach.

To assess the feasibility of the DHTS devices and the motor complications diary, we will assess whether the intended data was collected; specifically contextual data, self-reported medication adherence data, and mobility data for the smartphone, smartwatch, and IMU, respectively; and motor complications data for the motor complications diary (49). A summary of feasibility measures and their outcomes is included in Table 3.

Table 3. Summary of feasibility and outcomes extracted from DHTS and motor complications diary.

Device	Measure of feasibility	Outcomes extracted
**Smartphone**	Percentage of datasets collected over seven days and percentage of days missing	Number of steps taken outside the participant's home, per day
**Smartwatch**	Percentage of participants interacting with the smartwatch over seven day monitoring period	Number of interactions recorded on smartwatch

For peer review only - http://bmjopen.bmj.com/site/about/guidelines.xhtml

**IMU**	Percentage of datasets collected over seven day monitoring period	Walking speed and number of steps per day
**Motor complications diary**	Percentage of diaries returned and legible	Time spent in an ON/OFF state or experiencing dyskinesia

Only participants whose mobility data is collected for at least 3 days, will be included in analysis. If participants do not complete one of the study assessments (e.g., SPPB) or their data from the devices is not collected, their remaining data will still be included in analysis. Assuming missing data is at random, modern statistical techniques will be utilised to handle missing data and will be compared with a complete case approach (50).

Statistical analysis

Participants' descriptive, clinical, and Parkinson's specific characteristics, as well as data derived from the DHTS and motor complications diary data, will be analysed using descriptive statistics, including percentage and frequency, mean and standard deviation or median and interquartile range, as appropriate.

Functional data analysis and deep learning techniques (51) will be used to model and predict DMOs response to medication, and potential effects of contextual factors. DMO trends will be analysed between medication intake times and wearing-off effects will be evaluated via a customised scoring system. This scoring system will identify the wear off periods, grouping them relative to the strength of their effect. Nonlinear time series analysis will assess changes in DMOs between medication intakes (48, 52). Data collected using motor complications diaries will be used to optimise time series modelling of DMOs.

Should the data collected require an adjusted methodology, proposed statistical methods will be amended following completion of data collection.

Data management

All aspects of data management will adhere to the FAIR guiding principles, ensuring that data are findable, accessible, interoperable, and reusable (53), as well as being attributable, legible, contemporaneous, original, and accurate (ALCOA) (54). The CiC data management platform has been created on the open source, cloud-based platform e-Science Central (e-SC) (46, 47). This platform securely stores and shares data and has data processing capabilities.

This is a low risk, observational study which does not require a Data Monitoring Committee. The Data Management Group (SMG) will be responsible for managing data sharing requests, and upon data requests will meet with external researchers. This protocol received input from Parkinson's and clinical experts (LR, AY); technical experts (SDD, LA, JSQ) gave their input on data collection methods, outcomes, and eligibility criteria.

Data collection, storage, and archiving

To maintain confidentiality, participant data will be collected in a coded, anonymised manner using unique identification codes. Data will be directly uploaded onto the e-SC CiC platform or via third-party platforms (e.g., weather data). All researchers will be trained to correctly enter and upload data to relevant platforms. All data collected on paper case report forms will be signed, dated, scanned, and uploaded onto a secure network drive with restricted access. The e-SC CiC platform will be implemented using secure Amazon Web Services located within the European Union. The clinical site will keep original records of all paper forms in secure conditions until the study is completed. Long term data storage will be in

For peer review only - http://bmjopen.bmj.com/site/about/guidelines.xhtml

accordance with the Newcastle Joint Office policy for archiving, with data security arrangements made in accordance with Newcastle upon Tyne NHS Foundation Trust policies. The clinical site will be responsible for archiving study documents for a period which is in keeping with the institutional or national guidelines pertaining to the site. Detailed information on the data transfer pipeline is summarised in supplementary material 6.

Ethics and dissemination

This single centre study is sponsored by the Newcastle upon Tyne Hospitals NHS Foundation Trust (NuTH) UK. Ethical approval was obtained from the London - 142 Westminster Research Ethics Committee (REC reference: 21/PR/0469). The study will be conducted in accordance with Good Clinical Practice standards, the declaration of Helsinki, and The European Code of Conduct for Research Integrity (55). This study will be included in the NIHR Clinical Network Portfolio and has been registered in the ISRCTN registry (ISRCTN number: 13156149). Informed consent will be obtained by a trained researcher. The NHS indemnity scheme and Newcastle University are liable for harm to participants arising from the management and conduct of the study, and from its design, respectively.

As this is an observational study, there is no adverse event reporting planned. Should any Serious Adverse Events (SAEs) which are related to the administration of the research procedure occur, the sponsor and Chief Investigator will be informed. On behalf of the sponsor, the Newcastle Joint Research Office Governance Team will be responsible for assessing protocol deviations, serious breaches, and violations, as well as reporting Serious Breaches to the REC. The Chief Investigator is responsible for reporting protocol deviations and violations. Any protocol amendments will be communicated to the sponsor with all required amendment documents completed. The sponsor will be required to agree and accept any modifications, before amendments can be submitted to the NHS REC for their approval.

Dissemination policy

Newsletters will be sent to participants to inform them of study outcomes. Research will be disseminated through peer reviewed scientific journals, internal reports, conference presentations, and relevant websites. We will follow the International Committee of Medical Journal Editors recommendations for authorship eligibility.

Access to data

We plan to indefinitely maintain, the complete, anonymised dataset on the e-SC platform. On study completion, this dataset will be made available to the wider research community; upon request and will be shared with approved collaborators nationally and internationally for peer review, scientifically sound studies. Participants will have the right to access a copy of their data or request its removal following an appropriate request. These rights will terminate following dataset anonymisation and after the study key code is destroyed. The lead clinical investigator has overall responsibility for study execution and can access the full dataset. Data analysts, who are responsible for implementation of data protocol, will have access to the full-anonymised dataset. Statisticians will only have access to the full-anonymised datasets and will not have access to raw data files. Access to study data will be given to authorised representatives from the Sponsor, host institution, and regulatory authorities to allow study related audits, monitoring and inspections – in line with participant consent.

Study status

The first phase of recruitment has finished, with data collected in 30 PwP. Recruitment for the second phase of the study will start in February 2023 and will continue until June 2024 or until the desired sample size has been reached.

For peer review only - http://bmjopen.bmj.com/site/about/guidelines.xhtml

Footnotes

Author contributions

Study design: SDD, LA, AY, LR. Ethical approval and clinical oversight: SDD, LA, AY, LR. Manuscript initial drafting: EP, HD. Figures and tables preparation: EP, HD. Manuscript revision: EP, HD, SDD, LA. Intellectual contribution: EP, HD, HB, FC, NI, JE, MN, JQS, AY, LR, LA, SDD. All authors have provided critical intellectual input during manuscript revision. All authors have reviewed and approved the submitted manuscript. The corresponding author attests that all listed authors meet authorship criteria and that no others meeting the criteria have been omitted.

Funding

The study is co-funded by the Medical Research Council (MRC) Newcastle University (MC_PC_19047, NU-002884), the United Kingdom Research Innovation, Engineering and Physical Sciences Research Council (UKRI EPSRC) and the National Institute of Health Research (NIHR) (Grant Ref: EP/W031590/1, "Transforming care and health at home and enabling independence"). This study was also supported by the Mobilise-D project which has received funding from the Innovative Medicines Initiative 2 Joint Undertaking, under the grant agreement No. 820820. This JU receives support from the European Union's Horizon 2020 research and innovation program and the European Federation of Pharmaceutical Industries and Associations (EFPIA).

The research was supported by the National Institute for Health and Care Research (NIHR) Newcastle Biomedical Research Centre based at The Newcastle upon Tyne Hospitals NHS Foundation Trust, Newcastle University and the Cumbria, Northumberland and Tyne and Wear (CNTW) NHS Foundation Trust.

The research was also supported by NIHR Newcastle Clinical Research Facility (CRF) Infrastructure funding. All opinions are those of the authors, not the funders. Views expressed are those of the authors and do not necessarily reflect those of the NHS, the NIHR, UKRI EPSRC, Department of Health, the IMI, the European Union, the EFPIA or Associated Partners.

Competing interests

FC was CEO and shareholder of Aeqora Ltd. JE and MN are employees of McRoberts. SDD reports consultancy activity with Hoffmann-La Roche Ltd. outside of this study.

Acknowledgments

The authors would like to acknowledge and thank all collaborators of this study. We gratefully acknowledge the contribution of our much-missed colleague Dr Richard Dodds, who sadly passed away before this study could be completed.

References

1. Studenski S, Perera S, Patel K, et al. Gait speed and survival in older adults. Jama. 2011;305(1):50-8. doi: 10.1001/jama.2010.1923

2. Shumway-Cook A, Ciol MA, Yorkston KM, et al. Mobility Limitations in the Medicare Population: Prevalence and Sociodemographic and Clinical Correlates. Journal of the American Geriatrics Society. 2005;53(7):1217-21. doi: https://doi.org/10.1111/j.1532-5415.2005.53372.x

3. Armstrong MJ, Okun MS. Diagnosis and Treatment of Parkinson Disease: A Review. Jama. 2020;323(6):548-60. doi: 10.1001/jama.2019.22360

4. Paker N, Bugdayci D, Goksenoglu G, et al. Gait speed and related factors in Parkinson's disease. J Phys Ther Sci. 2015;27(12):3675-9. doi: 10.1589/jpts.27.3675

For peer review only - http://bmjopen.bmj.com/site/about/guidelines.xhtml

5. Malek N, Grosset DG. Medication adherence in patients with Parkinson's disease. CNS Drugs. 2015;29(1):47-53. doi: 10.1007/s40263-014-0220-0
6. Daley DJ, Myint PK, Gray RJ, et al. Systematic review on factors associated with medication non-adherence in Parkinson's disease. Parkinsonism Relat Disord. 2012;18(10):1053-61. doi: 10.1016/j.parkreldis.2012.09.004
7. Kulkarni AS, Balkrishnan R, Anderson RT, et al. Medication adherence and associated outcomes in medicare health maintenance organization-enrolled older adults with Parkinson's disease. Movement Disorders. 2008;23(3):359-65. doi: https://doi.org/10.1002/mds.21831
8. Campbell F, Ashburn A, Thomas P, et al. An exploratory study of the consistency of balance control and the mobility of people with Parkinson's disease (PD) between medication doses. Clin Rehabil. 2003;17(3):318-24. doi: 10.1191/0269215503cr614oa
9. Araújo-Silva F, Santinelli FB, Felipe IIL, et al. Temporal dynamics of cortical activity and postural control in response to the first levodopa dose of the day in people with Parkinson's disease. Brain Res. 2022;1775:147727. doi: 10.1016/j.brainres.2021.147727
10. Ávila de Oliveira J, Bazán PR, de Oliveira CEN, et al. The effects of levodopa in the spatiotemporal gait parameters are mediated by self-selected gait speed in Parkinson's disease. Eur J Neurosci. 2021;54(11):8020-8. doi: 10.1111/ejn.15522
11. Troisi Lopez E, Minino R, Sorrentino P, et al. Sensitivity to gait improvement after levodopa intake in Parkinson's disease: A comparison study among synthetic kinematic indices. PLoS One. 2022;17(5):e0268392. doi: 10.1371/journal.pone.0268392
12. Wu Z, Xu H, Zhu S, et al. Gait Analysis of Old Individuals with Mild Parkinsonian Signs and Those Individuals' Gait Performance Benefits Little from Levodopa. Risk Manag Healthc Policy. 2021;14:1109-18. doi: 10.2147/rmhp.S291669
13. Curtze C, Nutt JG, Carlson-Kuhta P, et al. Levodopa Is a Double-Edged Sword for Balance and Gait in People With Parkinson's Disease. Movement Disorders. 2015;30(10):1361-70. doi: https://doi.org/10.1002/mds.26269
14. Galna B, Lord S, Burn DJ, et al. Progression of gait dysfunction in incident Parkinson's disease: Impact of medication and phenotype. Movement Disorders. 2015;30(3):359-67. doi: https://doi.org/10.1002/mds.26110
15. Bryant MS, Rintala DH, Hou JG, et al. Gait variability in Parkinson's disease: influence of walking speed and dopaminergic treatment. Neurol Res. 2011;33(9):959-64. doi: 10.1179/1743132811y.0000000044
16. Smulders K, Dale ML, Carlson-Kuhta P, et al. Pharmacological treatment in Parkinson's disease: Effects on gait. Parkinsonism Relat Disord. 2016;31:3-13. doi: 10.1016/j.parkreldis.2016.07.006
17. Liu Y, Zhang G, Tarolli CG, et al. Monitoring gait at home with radio waves in Parkinson's disease: A marker of severity, progression, and medication response. Sci Transl Med. 2022;14(663):eadc9669. doi: 10.1126/scitranslmed.adc9669
18. Atrsaei A, Corrà MF, Dadashi F, et al. Gait speed in clinical and daily living assessments in Parkinson's disease patients: performance versus capacity. NPJ Parkinsons Dis. 2021;7(1):24. doi: 10.1038/s41531-021-00171-0
19. Del Din S, Galna B, Godfrey A, et al. Analysis of Free-Living Gait in Older Adults With and Without Parkinson's Disease and With and Without a History of Falls: Identifying Generic and Disease-Specific Characteristics. J Gerontol A Biol Sci Med Sci. 2019;74(4):500-6. doi: 10.1093/gerona/glx254
20. Del Din S, Godfrey A, Rochester L. Validation of an Accelerometer to Quantify a Comprehensive Battery of Gait Characteristics in Healthy Older Adults and Parkinson's Disease: Toward Clinical and at Home Use. IEEE J Biomed Health Inform. 2016;20(3):838-47. doi: 10.1109/jbhi.2015.2419317

For peer review only - http://bmjopen.bmj.com/site/about/guidelines.xhtml

21. Zhang T, Dong L, Jing H, et al. Smartphone Applications in the Management of Parkinson's Disease in a Family Setting: An Opinion Article. Frontiers in Neurology. 2021;12. doi: 10.3389/fneur.2021.668953
22. Roth N, Wieland GP, Kuderle A, et al. Do We Walk Differently at Home? A Context-Aware Gait Analysis System in Continuous Real-World Environments. Annu Int Conf IEEE Eng Med Biol Soc. 2021;2021:1932-5. doi: 10.1109/embc46164.2021.9630378
23. Fritz S, Lusardi M. White paper: "walking speed: the sixth vital sign". J Geriatr Phys Ther. 2009;32(2):46-9. doi, 20039582
24. Dorsey ER, Bloem BR, Okun MS. A New Day: The Role of Telemedicine in Reshaping Care for Persons With Movement Disorders. Mov Disord. 2020;35(11):1897-902. doi: 10.1002/mds.28296
25. Joshi R, Bronstein JM, Keener A, et al. PKG Movement Recording System Use Shows Promise in Routine Clinical Care of Patients With Parkinson's Disease. Frontiers in Neurology. 2019;10. doi: 10.3389/fneur.2019.01027
26. Chen L, Cai G, Weng H, et al. More Sensitive Identification for Bradykinesia Compared to Tremors in Parkinson's Disease Based on Parkinson's KinetiGraph (PKG). Front Aging Neurosci. 2020;12:594701. doi: 10.3389/fnagi.2020.594701
27. Chan AW, Tetzlaff JM, Gøtzsche PC, et al. SPIRIT 2013 explanation and elaboration: guidance for protocols of clinical trials. Bmj. 2013;346:e7586. doi: 10.1136/bmj.e7586
28. Mikolaizak AS, Rochester L, Maetzler W, et al. Connecting real-world digital mobility assessment to clinical outcomes for regulatory and clinical endorsement-the Mobilise-D study protocol. PLoS One. 2022;17(10):e0269615. doi: 10.1371/journal.pone.0269615
29. COSMIN. COnsensus-based Standards for the selection of health Measurement INstruments. [Available from: https://www.cosmin.nl/.
30. Goetz CG, Tilley BC, Shaftman SR, et al. Movement Disorder Society-sponsored revision of the Unified Parkinson's Disease Rating Scale (MDS-UPDRS): scale presentation and clinimetric testing results. Mov Disord. 2008;23(15):2129-70. doi: 10.1002/mds.22340
31. Hoehn MM, Yahr MD. Parkinsonism: onset, progression and mortality. Neurology. 1967;17(5):427-42. doi: 10.1212/wnl.17.5.427
32. Jette AM, Haley SM, Coster WJ, et al. Late life function and disability instrument: I. Development and evaluation of the disability component. J Gerontol A Biol Sci Med Sci. 2002;57(4):M209-16. doi: 10.1093/gerona/57.4.m209
33. Tomlinson CL, Stowe R, Patel S, et al. Systematic review of levodopa dose equivalency reporting in Parkinson's disease. Mov Disord. 2010;25(15):2649-53. doi: 10.1002/mds.23429
34. Schade S, Mollenhauer B, Trenkwalder C. Levodopa Equivalent Dose Conversion Factors: An Updated Proposal Including Opicapone and Safinamide. Mov Disord Clin Pract. 2020;7(3):343-5. doi: 10.1002/mdc3.12921
35. Fried LP, Tangen CM, Walston J, et al. Frailty in Older Adults: Evidence for a Phenotype. The Journals of Gerontology: Series A. 2001;56(3):M146-M57. doi: 10.1093/gerona/56.3.M146
36. Nieuwboer A, Rochester L, Herman T, et al. Reliability of the new freezing of gait questionnaire: agreement between patients with Parkinson's disease and their carers. Gait Posture. 2009;30(4):459-63. doi: 10.1016/j.gaitpost.2009.07.108
37. Nasreddine ZS, Phillips NA, Bédirian V, et al. The Montreal Cognitive Assessment, MoCA: a brief screening tool for mild cognitive impairment. J Am Geriatr Soc. 2005;53(4):695-9. doi: 10.1111/j.1532-5415.2005.53221.x
38. Guralnik JM, Simonsick EM, Ferrucci L, et al. A Short Physical Performance Battery Assessing Lower Extremity Function: Association With Self-Reported Disability and Prediction of Mortality and Nursing Home Admission. Journal of Gerontology. 1994;49(2):M85-M94. doi: 10.1093/geronj/49.2.M85

For peer review only - http://bmjopen.bmj.com/site/about/guidelines.xhtml

39. Rabinovich RA, Louvaris Z, Raste Y, et al. Validity of physical activity monitors during daily life in patients with COPD. European Respiratory Journal. 2013;42(5):1205-15. doi: 10.1183/09031936.00134312
40. Lord S, Galna B, Rochester L. Moving forward on gait measurement: Toward a more refined approach. Movement Disorders. 2013;28(11):1534-43. doi: https://doi.org/10.1002/mds.25545
41. Del Din S, Godfrey A, Galna B, et al. Free-living gait characteristics in ageing and Parkinson's disease: impact of environment and ambulatory bout length. Journal of NeuroEngineering and Rehabilitation. 2016;13(1):46. doi: 10.1186/s12984-016-0154-5
42. Hickey A, Del Din S, Rochester L, et al. Detecting free-living steps and walking bouts: validating an algorithm for macro gait analysis. Physiol Meas. 2017;38(1):N1-n15. doi: 10.1088/1361-6579/38/1/n1
43. Morris R, Hickey A, Del Din S, et al. A model of free-living gait: A factor analysis in Parkinson's disease. Gait Posture. 2017;52:68-71. doi: 10.1016/j.gaitpost.2016.11.024
44. Mazzà C, Alcock L, Aminian K, et al. Technical validation of real-world monitoring of gait: a multicentric observational study. BMJ Open. 2021;11(12):e050785. doi: 10.1136/bmjopen-2021-050785
45. Ciravegna F, Gao J, Ireson N, et al. Active 10: Brisk Walking to Support Regular Physical Activity. Proceedings of the 13th EAI International Conference on Pervasive Computing Technologies for Healthcare; Trento, Italy: Association for Computing Machinery; 2019. p. 11–20.
46. Hiden H, Woodman S, Watson P, et al. Developing cloud applications using the e-Science Central platform. Philos Trans A Math Phys Eng Sci. 2013;371(1983):20120085. doi: 10.1098/rsta.2012.0085
47. Din SD, Hickey A, Woodman S, et al. Accelerometer-based gait assessment: Pragmatic deployment on an international scale. 2016 IEEE Statistical Signal Processing Workshop (SSP). 2016:1-5. doi: 10.1109/SSP.2016.7551794
48. Rehman RZU, Buckley C, Mico-Amigo ME, et al. Accelerometry-Based Digital Gait Characteristics for Classification of Parkinson's Disease: What Counts? IEEE Open J Eng Med Biol. 2020;1:65-73. doi: 10.1109/ojemb.2020.2966295
49. World Health Organization. Monitoring and Evaluating Digital Health Interventions: A Practical Guide to Conducting Research and Assessment.; 2016.
50. Kang H. The prevention and handling of the missing data. Korean J Anesthesiol. 2013;64(5):402-6. doi: 10.4097/kjae.2013.64.5.402
51. Shi JQ, T C. Gaussian process regression analysis for functional data: CRC Press; 2011.
52. Del Din S, Godfrey A, Mazzà C, et al. Free-living monitoring of Parkinson's disease: Lessons from the field. Mov Disord. 2016;31(9):1293-313. doi: 10.1002/mds.26718
53. The European Commission D-GfRI. H2020 Programme Guidelines on FAIR Data. [Available from: https://ec.europa.eu/research/participants/docs/h2020-funding-guide/cross-cutting-issues/open-access-data-management/data-management_en.htm.
54. Management in Horizon 2020. 2016 [Available from: https://ec.europa.eu/research/participants/data/ref/h2020/grants_manual/hi/oa_pilot/h2020-hi-oa-data-mgt_en.pdf.
55. Association WM. World Medical Association Declaration of Helsinki: Ethical Principles for Medical Research Involving Human Subjects. JAMA. 2013;310(20):2191-4. doi: 10.1001/jama.2013.281053

For peer review only - http://bmjopen.bmj.com/site/about/guidelines.xhtml

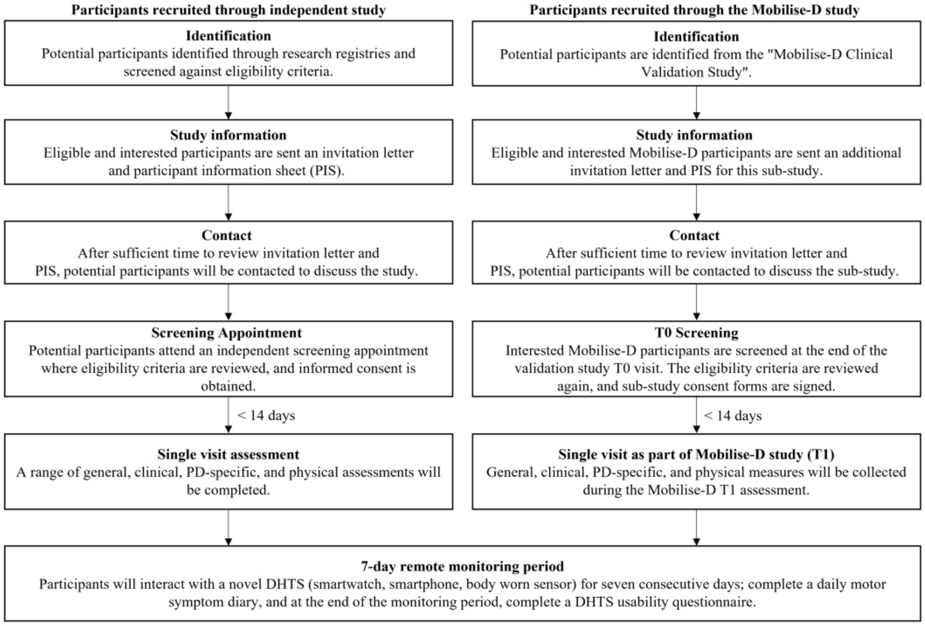

Figure 1. Flow chart of recruitment and screening procedure.

285x185mm (330 x 330 DPI)

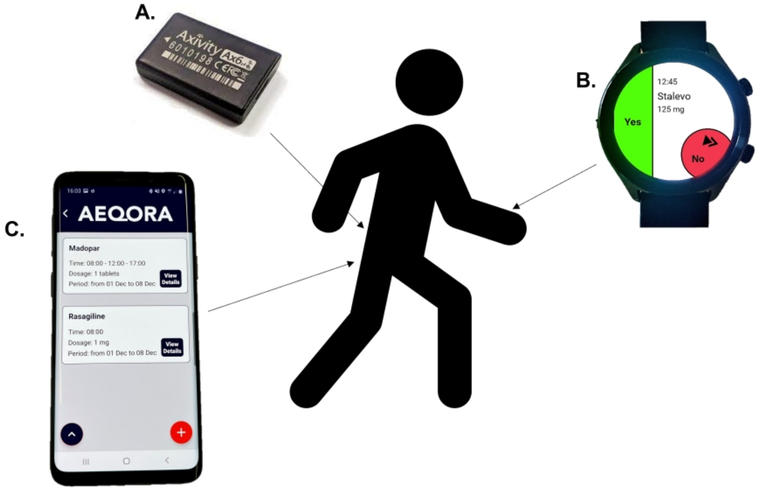

Figure 2 caption: ‘Figure 2. Digital Health Technology System (DHTS). (A) Inertial Measurement Unit (IMU), which monitors mobility. (B) Smartwatch which assesses self-reported medication adherence, by notifying participants when to take their medication and allowing them to log the time at which they took their medication. (C) Smartphone which collects contextual data.

306x218mm (330 x 330 DPI)

SPIRIT-Outcomes 2022 Checklist (for combined completion of SPIRIT 2013 and SPIRIT-Outcomes 2022 items)[a]

Section	Item No.	SPIRIT 2013 Item	SPIRIT-Outcomes 2022 item	Location Reported[b]
**Administrative information**				
Title	1	Descriptive title identifying the study design, population, interventions, and, if applicable, trial acronym	-	
Trial registration	2a	Trial identifier and registry name. If not yet registered, name of intended registry	-	
	2b	All items from the World Health Organization Trial Registration Data Set	-	
Protocol version	3	Date and version identifier	-	
Funding	4	Sources and types of financial, material, and other support	-	
Roles and responsibilities	5a	Names, affiliations, and roles of protocol contributors	-	
	5b	Name and contact information for the trial sponsor	-	
	5c	Role of study sponsor and funders, if any, in study design; collection, management, analysis, and interpretation of data; writing of the report; and the decision to submit the report for publication, including whether they will have ultimate authority over any of these activities	-	
	5d	Composition, roles, and responsibilities of the coordinating centre, steering committee, endpoint adjudication committee, data management team, and other individuals or groups overseeing the trial, if applicable (see Item 21a for data monitoring committee)	-	
**Introduction**				
Background and rationale	6a	Description of research question and justification for undertaking the trial, including summary of relevant studies (published and unpublished) examining benefits and harms for each intervention	-	
	6b	Explanation for choice of comparators	-	
Objectives	7	Specific objectives or hypotheses	-	

For peer review only

Section	Item No.	SPIRIT 2013 Item	SPIRIT-Outcomes 2022 item	Location Reported[b]
Trial design	8	Description of trial design including type of trial (eg, parallel group, crossover, factorial, single group), allocation ratio, and framework (eg, superiority, equivalence, noninferiority, exploratory)	-	
**Methods: Participants, interventions, and outcomes**				
Study setting	9	Description of study settings (eg, community clinic, academic hospital) and list of countries where data will be collected. Reference to where list of study sites can be obtained	-	
Eligibility criteria	10	Inclusion and exclusion criteria for participants. If applicable, eligibility criteria for study centres and individuals who will perform the interventions (eg, surgeons, psychotherapists)	-	
Interventions	11a	Interventions for each group with sufficient detail to allow replication, including how and when they will be administered (for specific guidance see TIDieR checklist and guide)	-	
	11b	Criteria for discontinuing or modifying allocated interventions for a given trial participant (eg, drug dose change in response to harms, participant request, or improving/worsening disease)	-	
	11c	Strategies to improve adherence to intervention protocols, and any procedures for monitoring adherence (eg, drug tablet return, laboratory tests)	-	
	11d	Relevant concomitant care and interventions that are permitted or prohibited during the trial	-	
Outcomes	12	Primary, secondary, and other outcomes, including the specific measurement variable (eg, systolic blood pressure), analysis metric (eg, change from baseline, final value, time to event), method of aggregation (eg, median, proportion), and time point for each outcome. Explanation of the clinical relevance of chosen efficacy and harm outcomes is strongly recommended	-	

For peer review only - http://bmjopen.bmj.com/site/about/guidelines.xhtml

Section	Item No.	SPIRIT 2013 Item	SPIRIT-Outcomes 2022 item	Location Reported[b]
	12.1		Provide a rationale for the selection of the domain for the trial's primary outcome	
	12.2		If the analysis metric for the primary outcome represents within-participant change, define and justify the minimal important change in individuals	
	12.3		If the outcome data collected are continuous but will be analyzed as categorical (method of aggregation), specify the cutoff values to be used	
	12.4		If outcome assessments will be performed at several time points after randomization, state the time points that will be used for analysis	
	12.5		If a composite outcome is used, define all individual components of the composite outcome	
Participant timeline	13	Time schedule of enrolment, interventions (including any run-ins and washouts), assessments, and visits for participants. A schematic diagram is highly recommended (see Figure)	-	
Sample size	14	Estimated number of participants needed to achieve study objectives and how it was determined, including clinical and statistical assumptions supporting any sample size calculations	-	
	14.1		Define and justify the target difference between treatment groups (eg, the minimal important difference)	
Recruitment	15	Strategies for achieving adequate participant enrolment to reach target sample size	-	
**Methods: Assignment of interventions (for controlled trials)**				
Allocation:				
Sequence generation	16a	Method of generating the allocation sequence (eg, computer-generated random numbers), and list of any factors for stratification. To reduce predictability of a random sequence, details of any planned restriction (eg, blocking) should be provided in a separate document that is unavailable to those who enrol participants or assign interventions	-	

For peer review only - http://bmjopen.bmj.com/site/about/guidelines.xhtml

Section	Item No.	SPIRIT 2013 Item	SPIRIT-Outcomes 2022 item	Location Reported[b]
Allocation concealment mechanism	16b	Mechanism of implementing the allocation sequence (eg, central telephone; sequentially numbered, opaque, sealed envelopes), describing any steps to conceal the sequence until interventions are assigned	-	
Implementation	16c	Who will generate the allocation sequence, who will enrol participants, and who will assign participants to interventions	-	
Blinding (masking)	17a	Who will be blinded after assignment to interventions (eg, trial participants, care providers, outcome assessors, data analysts), and how	-	
	17b	If blinded, circumstances under which unblinding is permissible, and procedure for revealing a participant's allocated intervention during the trial	-	
**Methods: Data collection, management, and analysis**				
Data collection methods	18a	Plans for assessment and collection of outcome, baseline, and other trial data, including any related processes to promote data quality (eg, duplicate measurements, training of assessors) and a description of study instruments (eg, questionnaires, laboratory tests) along with their reliability and validity, if known. Reference to where data collection forms can be found, if not in the protocol	-	
	18a.1		Describe what is known about the responsiveness of the study instruments in a population similar to the study sample	
	18a.2		Describe who will assess the outcome (eg, nurse, parent)	
	18b	Plans to promote participant retention and complete follow-up, including list of any outcome data to be collected for participants who discontinue or deviate from intervention protocols	-	

For peer review only - http://bmjopen.bmj.com/site/about/guidelines.xhtml

Section	Item No.	SPIRIT 2013 Item	SPIRIT-Outcomes 2022 item	Location Reported[b]
Data management	19	Plans for data entry, coding, security, and storage, including any related processes to promote data quality (eg, double data entry; range checks for data values). Reference to where details of data management procedures can be found, if not in the protocol	-	
Statistical methods	20a	Statistical methods for analysing primary and secondary outcomes. Reference to where other details of the statistical analysis plan can be found, if not in the protocol	-	
	20a.1		Describe any planned methods to account for multiplicity in the analysis or interpretation of the primary and secondary outcomes (eg, coprimary outcomes, same outcome assessed at multiple time points, or subgroup analyses of an outcome)	
	20b	Methods for any additional analyses (eg, subgroup and adjusted analyses)	-	
	20c	Definition of analysis population relating to protocol non-adherence (eg, as randomised analysis), and any statistical methods to handle missing data (eg, multiple imputation)	-	
**Methods: Monitoring**				
Data monitoring	21a	Composition of data monitoring committee (DMC); summary of its role and reporting structure; statement of whether it is independent from the sponsor and competing interests; and reference to where further details about its charter can be found, if not in the protocol. Alternatively, an explanation of why a DMC is not needed	-	
	21b	Description of any interim analyses and stopping guidelines, including who will have access to these interim results and make the final decision to terminate the trial	-	
Harms	22	Plans for collecting, assessing, reporting, and managing solicited and spontaneously reported adverse events and other unintended effects of trial interventions or trial conduct	-	

For peer review only - http://bmjopen.bmj.com/site/about/guidelines.xhtml

Section	Item No.	SPIRIT 2013 Item	SPIRIT-Outcomes 2022 item	Location Reported[b]
Auditing	23	Frequency and procedures for auditing trial conduct, if any, and whether the process will be independent from investigators and the sponsor	-	
**Ethics and dissemination**				
Research ethics approval	24	Plans for seeking research ethics committee/institutional review board (REC/IRB) approval	-	
Protocol amendments	25	Plans for communicating important protocol modifications (eg, changes to eligibility criteria, outcomes, analyses) to relevant parties (eg, investigators, REC/IRBs, trial participants, trial registries, journals, regulators)	-	
Consent or assent	26a	Who will obtain informed consent or assent from potential trial participants or authorised surrogates, and how (see Item 32)	-	
	26b	Additional consent provisions for collection and use of participant data and biological specimens in ancillary studies, if applicable	-	
Confidentiality	27	How personal information about potential and enrolled participants will be collected, shared, and maintained in order to protect confidentiality before, during, and after the trial	-	
Declaration of interests	28	Financial and other competing interests for principal investigators for the overall trial and each study site	-	
Access to data	29	Statement of who will have access to the final trial dataset, and disclosure of contractual agreements that limit such access for investigators	-	
Ancillary and post-trial care	30	Provisions, if any, for ancillary and post-trial care, and for compensation to those who suffer harm from trial participation	-	
Dissemination policy	31a	Plans for investigators and sponsor to communicate trial results to participants, healthcare professionals, the public, and other relevant groups (eg, via publication, reporting in results databases, or other data sharing arrangements), including any publication restrictions	-	
	31b	Authorship eligibility guidelines and any intended use of professional writers	-	

For peer review only - http://bmjopen.bmj.com/site/about/guidelines.xhtml

Section	Item No.	SPIRIT 2013 Item	SPIRIT-Outcomes 2022 item	Location Reported[b]
	31c	Plans, if any, for granting public access to the full protocol, participant-level dataset, and statistical code	-	
**Appendices**				
Informed consent materials	32	Model consent form and other related documentation given to participants and authorised surrogates	-	
Biological specimens	33	Plans for collection, laboratory evaluation, and storage of biological specimens for genetic or molecular analysis in the current trial and for future use in ancillary studies, if applicable	-	

[a]It is strongly recommended that this checklist be read in conjunction with the SPIRIT (Standard Protocol Items: Recommendations for Interventional Trials) Statement paper for important clarification on the items. Amendments to the protocol should be tracked and dated. The SPIRIT checklist is copyrighted by the SPIRIT Group under the Creative Commons "Attribution-NonCommercial-NoDerivs 3.0 Unported" license and is reproduced with permission.
[b]Indicates page numbers and/or manuscript location: to be completed by authors.

For peer review only

For peer review only - http://bmjopen.bmj.com/site/about/guidelines.xhtml

The Newcastle upon Tyne Hospitals
NHS Foundation Trust

Principal Investigator: Dr Silvia Del Din

Participant Identification Number:

Participant Consent Form

Confidence in Concept (CiC) - Translating digital healthcare to enhance clinical management: evaluating the effect of medication on mobility in people with Parkinson's disease (PD).

We are inviting you to take part in a medical research study.
Please make sure you have read the accompanying Participant Information Sheet which explains why we are doing this research and what we are asking you to do.
Please ask a member of the research team if there is anything that is not clear or if you would like more information.
It is entirely up to you if would like to take part or not.

For peer review only - http://bmjopen.bmj.com/site/about/guidelines.xhtml

The Newcastle upon Tyne Hospitals
NHS Foundation Trust

	Please initial box
I confirm that I have read and understand the Participant Information Sheet (version and date ……………………) for the above study. I have had the opportunity to consider the information, ask questions and have had these answered satisfactorily.	
I understand that my participation is voluntary and that I am free to withdraw at any time without giving any reason, without my medical care or legal rights being affected.	
I understand that relevant sections of my medical notes and data collected during the study may be looked at by individuals from local clinical care team, from Newcastle University, from regulatory authorities or from the NHS Trust, where it is relevant to my taking part in this research. I give permission for these individuals to have access to my records.	
I understand that the anonymised information collected about me will be stored and used to support other research in the future, and may be shared anonymously with other researchers.	
In the event that a new potential medical problem is found as a result of my participation, I agree that the research team may inform my GP or relevant medical professional about this following a discussion with myself.	
I understand that I will be provided with a wearable technology system comprising of an activity monitor, a smartwatch and a smartphone which will be worn and used for seven days following the visit.	
I agree to participate in this study.	

____________________	____________________	____________________
Name of Participant	Date	Signature

____________________	____________________	____________________
Name of Person Taking Consent	Date	Signature

For peer review only - http://bmjopen.bmj.com/site/about/guidelines.xhtml

Please ensure that a copy is offered to the participant or relative/carer and record whether this offer was accepted/ declined.

Copy given [ ]	Copy declined [ ]

Original to be filed in the Trial Master File. Copy to be included in the participant medical records.

For peer review only

For peer review only - http://bmjopen.bmj.com/site/about/guidelines.xhtml

The Newcastle upon Tyne Hospitals
NHS Foundation Trust

Principal Investigator: Dr Silvia Del Din

Participant Identification Number:

Participant Consent Form Consent Form for Mobilise-D – Clinical Validation Sub-Study

Confidence in Concept (CiC) - Translating digital healthcare to enhance clinical management: evaluating the effect of medication on mobility in people with Parkinson's disease (PD).

As a participant in the Mobilise-D - Clinical Validation Study, we are asking you to volunteer to take part in additional data collection for a sub-study.
Please make sure you have read the accompanying Participant Information Sheet which explains why we are doing this research and what we are asking you to do.
Please ask a member of the research team if there is anything that is not clear or if you would like more information.
It is entirely up to you if would like to take part or not in this additional data collection.

For peer review only

For peer review only - http://bmjopen.bmj.com/site/about/guidelines.xhtml

CiC – Effect of medication on mobility in people with PD
Consent Form V2.1 - 14 May 2021
IRAS ID: 295771

NHS
The Newcastle upon Tyne Hospitals
NHS Foundation Trust

	Please initial box
I confirm that I have read and understand the Participant Information Sheet (version and date ……………………) for the above study. I have had the opportunity to consider the information, ask questions and have had these answered satisfactorily.	
I understand that my participation is voluntary and that I am free to withdraw at any time without giving any reason, without my medical care or legal rights being affected.	
I understand that relevant sections of my medical notes and data collected during the study may be looked at by individuals from local clinical care team, from Newcastle University, from regulatory authorities or from the NHS Trust, where it is relevant to my taking part in this research. I give permission for these individuals to have access to my records.	
I understand that the anonymised information collected about me will be stored and used to support other research in the future, and may be shared anonymously with other researchers.	
In the event that a new potential medical problem is found as a result of my participation, I agree that the research team may inform my GP or relevant medical professional about this following a discussion with myself.	
I understand that as part of the Mobilise-D Clinical Validation Study I will be provided with an activity monitor which will be worn for seven days following the baseline visit and that, for this additional data collection, I will be also provided with a smartwatch and a smartphone which will be used during the same seven day period.	
I agree to participate in this sub-study and additional data collection.	

Name of Participant	Date	Signature
Name of Person Taking Consent	Date	Signature

For peer review only - http://bmjopen.bmj.com/site/about/guidelines.xhtml

NHS
The Newcastle upon Tyne Hospitals
NHS Foundation Trust

Please ensure that a copy is offered to the participant or relative/carer and record whether this offer was accepted/ declined.

Copy given [ ]	Copy declined [ ]

Original to be filed in the Trial Master File. Copy to be included in the participant medical records.

For peer review only

For peer review only - http://bmjopen.bmj.com/site/about/guidelines.xhtml

CiC – Effect of medication on mobility in people with PD

Version 1.1; 14 May 2021; IRAS ID: 295771

Subject Initial |___||___|
F S

Subject ID |___|___|___|___|___|

**Day/ Time**	**6:00 7:00**	**7:00 8:00**	**8:00 9:00**	**9:00 10:00**	**10:00 11:00**	**11:00 12:00**	**12:00 13:00**	**13:00 14:00**	**14:00 15:00**	**15:00 16:00**	**16:00 17:00**	**17:00 18:00**	**18:00 19:00**	**19:00 20:00**	**20:00 21:00**	**21:00 22:00**	Comments
Monday																	
Tuesday																	
Wednesday																	
Thursday																	
Friday																	
Saturday																	
Sunday																	

*Split cells indicate the "half an hour" – e.g. 6.30

Example: Off status ("O") between 6.30 and 7.00 and dyskinesia ("D") between 7.00 and 7.30 on Monday

**Day/ Time**	**6:00 7:00**		**7:00 8:00**		**8:00 9:00**	
Monday		O	D			

For peer review only

CiC – Effect of medication on mobility in people with PD

Version 1.1; 14 May 2021; IRAS ID: 295771

**Subject Initial**	**Subject ID**
|___||___| F S	|___|___|___|___|___|

Usability questionnaire

For peer review only

Subject Initials **Subject ID**

|__||__| |__|__|__|__|__|

F S

English versions of questionnaires

Rabinovich

Please select a response for each of these questions that best reflects your experiences and opinions of the Wearable Technology System (Wearable device, smartwatch and smartphone).

Section A

How much trouble did you have getting started with the Wearable Technology System?

(NOTE: 'getting started with' refers to the first few hours of wear/use in the week. It is how the participant felt while using/wearing it initially at the start of the week)

O No trouble to start up

O Sometimes trouble to start up

O Regularly caused trouble to start up

O Always trouble to start up

O I had to call the centre to get help in starting-up

The Wearable Technology System was easy to put on/take off

O Yes this was very easy

O This worked just fine

O I found it somewhat difficult

O I found it difficult

O I was unable to manage this on my own

I experienced technical problems with the Wearable Technology System

O All the time

O Frequently

O Sometimes

O Seldom

O Never

For peer review only

Subject Initials |__||__|
F S

Subject ID |__|__|__|__|__|

The Wearable Technology System interfered with my normal activities

O All the time

O Frequently

O Sometimes

O Occasionally

O Never

I felt comfortable wearing the Wearable Technology System

O All the time

O Frequently

O Sometimes

O Occasionally

O Never

I felt embarrassed wearing the Wearable Technology System

O All the time

O Frequently

O Sometimes

O Occasionally

O Never

The instructions on how to use the Wearable Technology System were clear

O Strongly disagree

O Disagree

O Neutral

O Agree

O Strongly agree

For peer review only

CiC – Effect of medication on mobility in people with PD

Subject Initials |__||__|
F S

Subject ID |__|__|__|__|__|

Using the Wearable Technology System on a daily basis was easy

O Strongly disagree

O Disagree

O Neutral

O Agree

O Strongly agree

The Wearable Technology System was bulky/heavy.

O Yes , very much so

O Yes much

O Not particularly

O Not at all

O No opinion

The Wearable Technology System (Wearable device) bothered me in the bed.

O Yes , very much so

O Yes much

O Not particularly

O Not at all

O No opinion, I did not wear the monitor at night

I felt my privacy was invaded by the Wearable Technology System

O Strongly disagree

O Disagree

O Neutral

O Agree

O Strongly agree

For peer review only

Subject Initials |__||__|
F S

Subject ID |__|__|__|__|__|__|

If my doctor would like to use the Wearable Technology System to assess my activity and medication adherence I would be willing to wear it and use it for

O Less than 1 day

O 2-4 days

O 1week

O Longer than 1 week.

O I would not mind wearing the monitor continuously (longer than 1 month)

Section B

We want to ask you to provide a final score for the Wearable Technology System.

All things considered can you give the device a score from 0% to 100%, where 0 means the worst possible system and 100% means the ideal system in your opinion.

My score for Wearable Technology System: .... /100

In the section below we want to give you the opportunity to give other comments on the Wearable Technology System and its component (wearable device (lower back), smartwatch and smartphone).

Wearable device:

Smartwatch:

Smartphone:

WT as a whole:

I experienced the following problems with the Wearable Technology System:

Wearable device:

Smartwatch:

Smartphone:

WT as a whole:

I liked these features of the Wearable Technology System in particular:

Wearable device:

Smartwatch:

Smartphone:

WT as a whole:

For peer review only

For peer review only - http://bmjopen.bmj.com/site/about/guidelines.xhtml

Supplementary material 6. Table summarising the standardised and secure data transfer pipeline.

**Data type**	**Data pipeline**
**Clinical and Demographic data**	Data will be collected on paper forms, which will be scanned and uploaded to a secured password protected University drive. Processed data will be entered onto the database. Paper forms will be stored in the Gait Laboratory at the Clinical Ageing Research Unit, with access restricted to the laboratory research team.
**Mobility data from IMU**	When using the Axivity AX6 sensor, raw data will be directly uploaded to the data management platform on e-SC from the clinical site.
**Self-reported medication adherence data (smartwatch and smartphone)**	Data that are logged on the smartphone will be transferred to the University of Sheffield (USFD) servers. Processed data (e.g., acknowledged medication intake times) will then be transferred from USFD servers and uploaded to eSC platform via a secured system (e.g. API or web portal).
**Contextual data from smartphone**	Data will be transferred from the smartphone to the USFD servers. Processed data (annotations) will be transferred from USFD servers and uploaded to the eSC platform via a secured system (e.g. API or web portal).
**Usability questionnaire, open-text questions, and motor complications diary**	Usability questionnaires, responses to open-text questions, and diary data will be collected with paper forms. These forms will be scanned and uploaded to a secured password protected University drive. Processed data will be entered onto a database.

For peer review only
